# Alternative splicing diversifies the skeletal muscle transcriptome during prolonged spaceflight

**DOI:** 10.1186/s13395-022-00294-9

**Published:** 2022-05-31

**Authors:** Mason Henrich, Pin Ha, Yuanyuan Wang, Kang Ting, Louis Stodieck, Chia Soo, John S. Adams, Rene Chun

**Affiliations:** 1 Department of Molecular, Cell & Developmental Biology, University of California, 615 Charles E Young Dr S Room 446, Los Angeles, CA 90095 USA; 2 Department of Surgery, Division of Plastic and Reconstructive Surgery, University of California, Los Angeles, USA; 3 Bioinformatics IDP, University of California, Los Angeles, USA; 4 Forsyth Institute, Cambridge, MA USA; 5 Department of Aerospace Engineering Sciences, BioServe Space Technologies, University of Colorado, Boulder, USA; 6 Department of Orthopaedic Surgery, University of California, Los Angeles, USA

**Keywords:** Microgravity, Spaceflight, Alternative splicing, Transcriptome, Skeletal muscle

## Abstract

**Background:**

As the interest in manned spaceflight increases, so does the requirement to understand the transcriptomic mechanisms that underlay the detrimental physiological adaptations of skeletal muscle to microgravity. While microgravity-induced differential gene expression (DGE) has been extensively investigated, the contribution of differential alternative splicing (DAS) to the plasticity and functional status of the skeletal muscle transcriptome has not been studied in an animal model. Therefore, by evaluating both DGE and DAS across spaceflight, we set out to provide the first comprehensive characterization of the transcriptomic landscape of skeletal muscle during exposure to microgravity.

**Methods:**

RNA-sequencing, immunohistochemistry, and morphological analyses were conducted utilizing total RNA and tissue sections isolated from the gastrocnemius and quadriceps muscles of 30-week-old female BALB/c mice exposed to microgravity or ground control conditions for 9 weeks.

**Results:**

In response to microgravity, the skeletal muscle transcriptome was remodeled via both DGE and DAS. Importantly, while DGE showed variable gene network enrichment, DAS was enriched in structural and functional gene networks of skeletal muscle, resulting in the expression of alternatively spliced transcript isoforms that have been associated with the physiological changes to skeletal muscle in microgravity, including muscle atrophy and altered fiber type function. Finally, RNA-binding proteins, which are required for regulation of pre-mRNA splicing, were themselves differentially spliced but not differentially expressed, an upstream event that is speculated to account for the downstream splicing changes identified in target skeletal muscle genes.

**Conclusions:**

Our work serves as the first investigation of coordinate changes in DGE and DAS in large limb muscles across spaceflight. It opens up a new opportunity to understand (i) the molecular mechanisms by which splice variants of skeletal muscle genes regulate the physiological adaptations of skeletal muscle to microgravity and (ii) how small molecule splicing regulator therapies might thwart muscle atrophy and alterations to fiber type function during prolonged spaceflight.

**Supplementary Information:**

The online version contains supplementary material available at 10.1186/s13395-022-00294-9.

## Background

With the rapidly expanding scientific and commercial interests in space exploration, an increase in long-term human ventures into space is inevitable but not without risk. Even before man entered this new frontier in 1961, astronomers and clinicians alike cautioned that “it will not be engineering problems but rather the limits of the human frame that will make the final decision as to whether manned spaceflight will eventually become and remain a reality” [1]. Therefore, biomedical researchers have worked tirelessly to keep pace with breakthroughs in aerospace engineering over the last half-century by characterizing the impact of spaceflight on the human body and studying interventions to mitigate the adverse influence of sustained weightlessness.

For example, prolonged disuse of skeletal muscle, often referred to as mechanical unloading, precipitates muscle atrophy in microgravity environments, characterized by a loss of muscle mass and strength [2]. Similar microgravity-associated muscle phenotypes have been identified in the limb muscles of mice [3], rats [4], and monkeys [5] exposed to microgravity as well as sedentary human populations such as the elderly [6] and handicapped [7]. While countermeasures, chief among them being regular exercise, are beneficial [8], these interventions fail to completely prevent microgravity-induced atrophy [9]. Ultimately, after only 1 month of exposure to microgravity, skeletal muscle can decrease up to 20% in mass and 30% in strength [10]. This loss of mass and strength prevents astronauts from performing mission tasks and puts them at increased risk of injury upon return to higher gravity conditions [11].

While aggressive muscle conditioning and rehabilitation on Earth can eventually restore muscle mass and strength, this process can take anywhere from a few months to 4 years, with muscle type-specific differences in rehabilitation time attributed to compositional and functional distinctions between different muscles [12]. Compositionally, skeletal muscle is defined by its myosin heavy chain (MyHC) expression pattern, with slow-twitch muscles predominantly expressing MyHC I isoforms and fast-twitch muscles predominantly expressing MyHC II isoforms [13]. Functionally, skeletal muscle is defined by its role in controlling movement. Most microgravity research has focused on skeletal muscles that control sagittal plane movements, including flexion (bending of a joint) and extension (straightening of a joint) [14]. The gastrocnemius and the quadriceps are the muscles of the mouse hind limb studied here. The gastrocnemius expresses both MyHC I and MyHC II isoforms and functions as an ankle extensor and knee flexor, while the quadriceps predominantly expresses the MyHC II isoform and functions as a knee extensor and hip flexor [14].

The composition and function of skeletal muscle dictates the nature and extent of its response to microgravity. For example, extended spaceflight induces more atrophy in slow-twitch fibers than in fast-twitch fibers and more in primary extensors than in primary flexors, with the atrophic response beginning in the largest fibers within a muscle [15, 16]. Although muscle atrophy is the most prominent physiological adaptation of skeletal muscle to microgravity, skeletal muscles are also subject to muscle type-specific fiber type alterations. As is implied in their nomenclature, fast-twitch fibers are responsible for dynamic movement while slow-twitch fibers support low-level, sustained activity [14]. Therefore, reliance on the dynamic component of motor function in microgravity necessitates an adaptive increase in fast-twitch fiber content at the expense of existing slow-twitch fibers [17, 18].

The development of high-throughput sequencing technologies has spurred extensive interest in elucidating the transcriptomic underpinnings of microgravity-induced phenotypes. Most interest has been paid to the transcriptomic effects at the level of differential gene expression (DGE). Recent transcriptome analyses of skeletal muscle in mice [19–21] have annotated microgravity-induced DGE of gene networks related to contractile machinery, calcium homeostasis, muscle development, cellular metabolism, inflammatory/oxidative stress response, and mitochondrial function. One landmark study, the National Aeronautics and Space Administration (NASA) Twins Study [22], identified DGE between monozygotic twins that were exposed to spaceflight or ground control conditions for one year. While this study occurred in the context of peripheral blood mononuclear cells and FACS-sorted immune cells, some transcriptomic changes persisted up to 6 months after return to Earth, suggesting that transcriptome remodeling due to spaceflight is not entirely transient.

However, a focus restricted to the level of DGE fails to account for the actions of alternative splicing (AS) on the host’s transcriptome. RNA-binding protein (RBP)-mediated AS accounts for the multi-fold increase in the diversity of translatable mRNA isoforms over what can be accounted for by the approximately 30,000 genes in the human genome [23]. In fact, approximately 95% of human genes have been found to exhibit alternatively spliced isoforms [24], most attributable to the best characterized and most prevalent AS types: skipped exon (SE) and mutually exclusive exon (MXE) events [25]. The role of AS in skeletal muscle on Earth has been well-annotated, including regulation of myogenesis [26–28], cell type-specific function [29–31], fiber-type-specific function [32], muscle contraction [33, 34], calcium handling [35–37], muscle atrophy [38, 39], and muscular dystrophy [40]. However, there have been no investigations of differential alternative splicing (DAS) in skeletal muscle during or following spaceflight. In fact, there has been only a single examination of microgravity-induced DAS, and that study focused on *Arabidopsis thaliana*, a species of flowering plant [41].

Here, we set out to characterize, for the first time in an animal model, diversification of the skeletal muscle transcriptome by DAS in microgravity. Total RNA and tissue sections were isolated from the gastrocnemius and quadriceps muscles of 30-week-old female BALB/c mice exposed to either microgravity or ground control conditions for a total of 9 weeks. Using RNA-sequencing (RNA-seq), immunohistochemistry, and morphological analyses, we aimed to characterize functionally significant DAS changes in non-differentially expressed skeletal muscle genes that describe a previously uninvestigated means of modifying the transcriptome in response to the physiologic demands of microgravity.

## Methods

### Experimental design and timeline

Twenty 30-week-old female BALB/c mice (Taconic Biosciences, NY) were used in this study. These animals were opportunistically obtained from a study originally designed to analyze changes in bone following microgravity exposure and test a novel therapeutic for osteoporosis. In the study presented here, only animals that received control therapy (subcutaneous phosphate-buffered saline (PBS) injections every two weeks over the total 9 weeks of experimentation) were used. The age, gender, and strain of our mice were chosen based on the design of the osteoporosis study. In regard to age, 30 weeks is around the timepoint at which mouse hind limb bone mineral density (BMD) reaches its peak and stabilizes until its eventual decline around the age of two years [42, 43]. Therefore, any changes in BMD observed after 9 weeks of spaceflight could be definitively attributed to the effects of microgravity rather than developmental and/or aging processes. In regard to sex, female mice are often favored over male mice in osteoporosis research as ovariectomy is the best-established, most clinically relevant model of postmenopausal osteoporosis [44]. Finally, regarding strain, BALB/c mice are a common model for osteoporosis therapy testing because BALB/c females respond optimally to ovariectomy and hypogonadism compared to other strains [45] and BALB/c males demonstrate more prevalent glucocorticoid-induced secondary osteoporosis than C57BL/6 males [46].

**Fig. 1:**
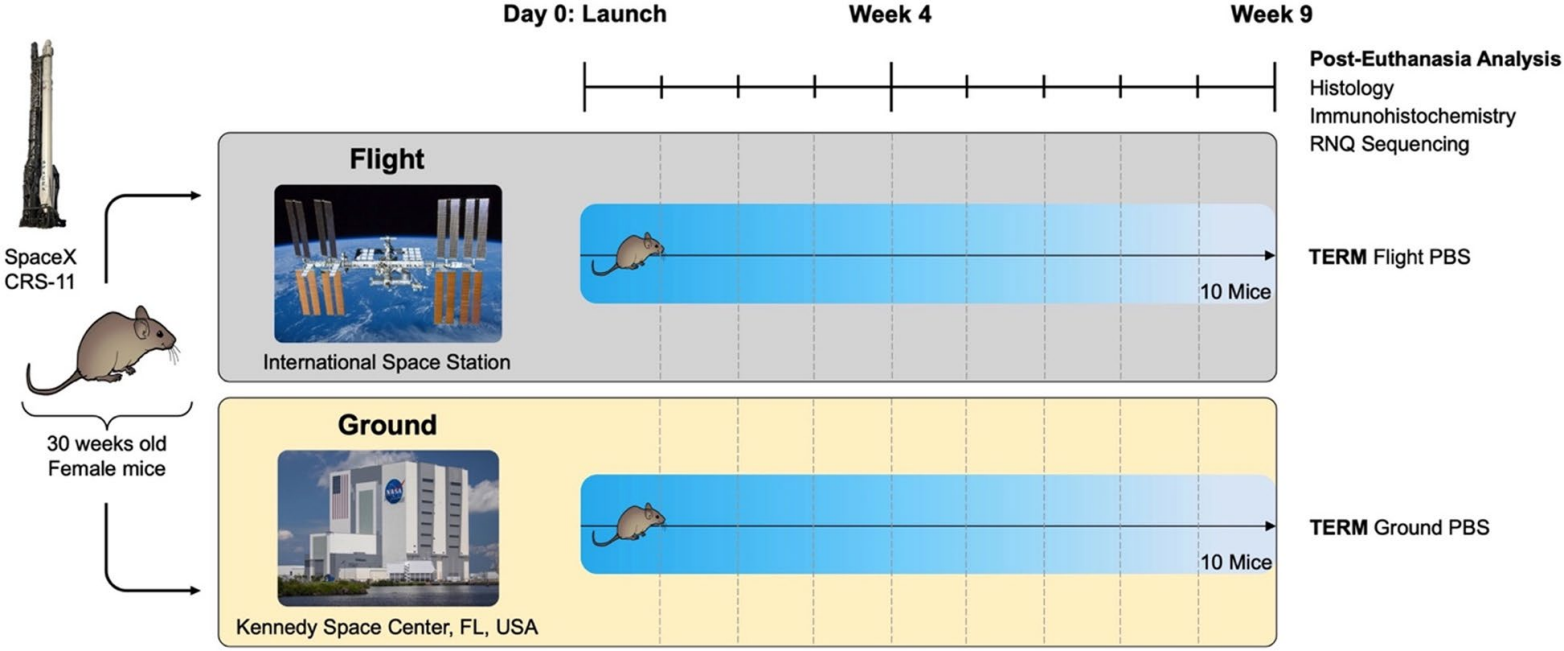
Schematic timeline of experimentation. Twenty 30-week-old female BALB/c mice were exposed to flight (ISS, *n* = 10) or ground (KSC, *n* = 10) conditions for 9 weeks. Post-euthanasia analyses included histology, immunohistochemistry, and RNA-sequencing. CRS, Commercial Resupply Service; TERM, terminal; PBS, phosphate-buffered saline-treated

All mice were housed at the Kennedy Space Center (KSC) in Florida, USA, before rocket launch and were randomly divided into ground control and flight groups (*n* = 10 per group). On June 3rd, 2017, flight mice were transported to the International Space Station (ISS) as part of SpaceX Commercial Resupply Service (CRS)-11 and kept on board the ISS for the full 9 weeks of experimentation, while ground control mice were kept at KSC for the same duration (Fig. 1). Ground control mice were housed in identical hardware (Rodent Research Hardware System, NASA Ames Research Center, https://www.nasa.gov/ames/research/space-biosciences/rodent-research-hardware) to that of flight mice and housed under matched environmental conditions (temperature, humidity, and carbon dioxide levels). Flight and ground control mice were provided ad lib access to water and specially developed NASA Nutrient Food Bars [47].

### Sample preparation

At the end of the study, all mice were humanely euthanized on board the ISS and at the KSC by trained astronauts or ground personnel, respectively. The right hind limb, with skin removed, was dissected at the hip and submerged in 10% neutral-buffered formalin followed 6 days later with a PBS wash and submersion in 70% ethanol for long-term storage. Right hind limbs were stored at room temperature until return for dissection of individual muscles for immunohistochemistry and morphology analyses. The remaining mouse carcasses were then frozen to − 80°C or colder. Samples from the flight condition returned to Earth on SpaceX CRS-12 on September 17th, 2017. All samples were transported to the University of California, Los Angeles (UCLA), on dry ice. Frozen carcasses were thawed on wet ice before tissue dissection. Skeletal muscles from the left hind limb of each carcass were individually dissected and preserved in *RNAlater* preservative solution (Invitrogen, Waltham, MA, USA) for RNA-seq following RNA extraction and purification.

### Immunohistochemistry and morphological analyses

Formalin-fixed, paraffin-embedded sections of mouse muscle (5-μm thickness) from the gastrocnemius and quadriceps were cut with a microtome and mounted on charged slides. Sections were either subjected to standard hematoxylin-eosin staining for overview or immunolabeled with the following anti-MyHC isoform antibodies: type I MyHC isoform (Abcam, Cat# ab11083); type II MyHC isoform (Abcam, Cat# ab51263). Sections were co-stained with an anti-laminin antibody (Abcam, Cat# ab11575) to allow measurement of fiber size. In all protocols, donkey anti-rabbit Alexa-488 conjugated secondary antibody (Abcam, Cat# ab150073) was used for laminin staining, donkey anti-mouse Alexa-594 conjugated secondary antibody (Abcam, Cat# ab150108) was used for type I MyHC antigen staining, and donkey anti-mouse Alexa-488 conjugated secondary antibody (Abcam, Cat# ab150105) was used for type II MyHC antigen staining. Photomicrographs were acquired using Olympus BX 51 and IX 71 microscopes equipped with Cell Sense digital imaging system (Olympus, Japan).

To assess the cross-sectional area (CSA) of the myofiber, digitized photographs were acquired from immunofluorescence sections stained with anti-laminin antibody and CSA was measured as described previously [48]. Briefly, each image of the cross-sectioned muscle bundle was outlined in ImageJ 1.45g (National Institutes of Health Image, https://imagej.nih.gov/nih-image/). Histology artifacts (e.g., section tears, wrinkles), anatomic structures that interfered with muscle fiber recognition (e.g., blood vessels, tendons, oblique fibers), and poorly detected muscle fibers were removed manually from analysis using the software exclusion tool. CSA of all remaining fibers was determined using a pixel to micrometer conversion factor estimated with a precision ruler (0.647 μm/pixel), and the average fiber area was reported automatically by the software. One entire mid-bundle section was quantified per mouse.

### RNA extraction and purification

Total RNA was isolated from mouse gastrocnemius (*n* = 3) and quadriceps (*n* = 3) of each experimental group (flight and ground control) using the acid guanidinium thiocyanate-phenol-chloroform extraction followed by silica membrane purification. Briefly, frozen tissue samples were minced into small pieces (1 mm × 1 mm × 1 mm). A homogeneous lysate was achieved by adding lysing buffer and gentle ultrasound vibration on ice. The tissue lysate was centrifuged and the supernatant was used for RNA extraction in phenol/chloroform. After phase separation, the aqueous layer was transferred and mixed with an equal volume of 70% ethanol. Total RNA was then extracted using RNeasy spin columns from the RNeasy Micro Kit (Qiagen, Hilden, Germany) according to the manufacturer’s protocol.

### RNA-sequencing analysis

For all 12 RNA samples, cDNA libraries were prepared by the UCLA Technology Center for Genomics and Bioinformatics (TCGB) following the Illumina stranded mRNA protocol. Libraries were then sequenced by the UCLA TCGB utilizing a HiSeq 3000 sequencer (Illumina Inc., San Diego, CA, USA), generating an average of 41.5 million single-end 50 base pair reads. The resulting RNA-seq reads were aligned to the mm10 *Mus musculus* genome (UCSC Genome Browser, https://genome.ucsc.edu/cgi-bin/hgGateway?db=mm10) reference using the STAR software [49]. Quality of the RNA-seq dataset was confirmed by read depth and mapping statistics (Fig. S1A). Read depth for all samples was at or above 30 million uniquely mapped reads, with the exception of one ground control quadriceps sample with 27.4 million uniquely mapped reads. The uniquely mapped read percentage for all samples was above 80%. Transcript abundance was measured directly from FASTQ files as TPM (transcripts per million) using kallisto [50] and summarized into gene expression matrix by R package “tximport” [51].

DAS events were detected and quantified as percent spliced in (PSI) values by rMATS-turbo [52] using junction reads (reads spanning the splicing junctions). Five DAS event types were annotated (Fig. S1B), including skipped exon (SE), alternative 5′ splice site (A5SS), alternative 3′ splice site (A3SS), mutually exclusive exons (MXE), and retained intron (RI). SE and MXE, the DAS event types focused on in this work, composed approximately 50% and 20% of all DAS events, respectively. Gene ontology (GO) analysis was performed to reveal enriched functional pathways affected by significant gene expression changes as well as alternative splicing changes using EnrichR [53–55].

### Statistical analysis

For immunohistochemistry and morphological analyses, statistical significance was performed with OriginPro 8 (Origin Lab Corp., Northampton, MA, USA) using an unpaired, two-tailed Student’s *t-*test. A value of *p* < 0.05 was considered to indicate a statistically significant difference. The statistical analyses were performed in consultation with the UCLA Statistical Biomathematical Consulting Service.

For annotation of statistically significant DGE, lowly expressed genes (TPM ≤ 5 in all samples) were filtered out before conducting differential analysis with DeSeq2 [56]. For each comparison, genes with an absolute log_2_ fold change > log_2_ 1.5 and a false discovery rate (FDR)-adjusted *p*-value < 0.05 were assumed to be differentially expressed genes.

For detection of alternative splicing events in the dataset, events with low junction read support (≤ 10 average junction reads, ≤ 10 total inclusion junction reads, or ≤ 10 total skipping junction reads over all 12 samples), or with extreme PSI value ranges (PSI ≤ 0.05 or ≥ 0.95 in all 12 samples) were excluded from downstream analysis. Differential splicing analysis was then performed using rMATS-turbo by comparing the replicate ground control samples and flight samples in each tissue (gastrocnemius and quadriceps). Differential splicing events were identified by the following criteria: (i) > 10 average junction reads (inclusion and skipping junction reads) in both groups; (ii) no extreme PSI values (PSI ≤ 0.05 or PSI ≥ 0.95 for all 6 samples in the comparison); (iii) FDR < 0.05; iv) absolute change in PSI (|ΔPSI|) > 0.05.

For annotation of enriched functional pathways affected by significant gene expression changes as well as alternative splicing changes, the five GO terms from each category (biological processes, molecular function, and cellular component) with the smallest adjusted *p*-values were included for reference. A value of adjusted *p* < 0.05 was considered to indicate a statistically significant difference.

## Results

### The size and fiber type composition of the gastrocnemius and quadriceps were differentially perturbed by prolonged spaceflight

**Fig. 2:**
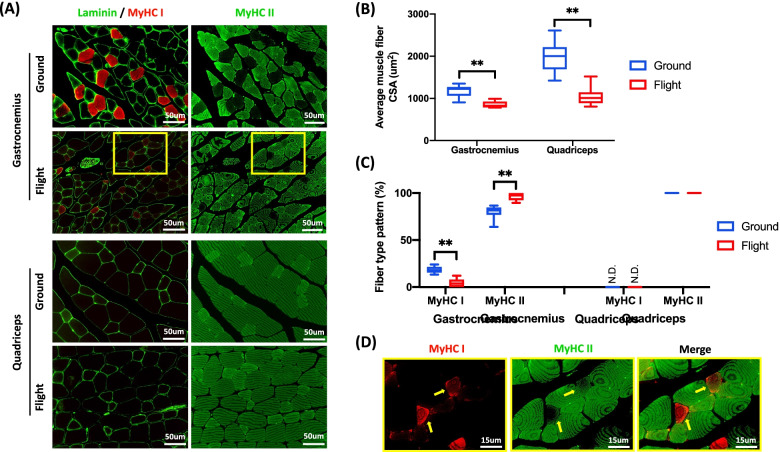
Physiological adaptations to microgravity are muscle type-specific. **A** Representative immunofluorescence images of the gastrocnemius and quadriceps stained for laminin and MyHC isoforms (*left panel* laminin, green; MyHC I, red) (*right panel* MyHC II, green) in ground control and flight mice. **B** Box-and-whisker plots quantify the myofiber CSA distribution (mean value, upper/lower quartiles, and maximum/minimum) in both gastrocnemius and quadriceps. Asterisks represent significance by two-tailed *t*-test (**p < 0.01). **C** Box-and-whisker plots quantify the muscle fiber type distribution (mean value, upper/lower quartiles, and maximum/minimum) in both gastrocnemius and quadriceps. Asterisks represent significance by two-tailed t-test (**p < 0.01). **D** Some fibers (yellow arrows) within the gastrocnemius of spaceflown mice were co-stained by both MyHC isoforms

On average, the CSA of muscle fibers composing the gastrocnemius decreased from 1170 ± 141 μm^2^ to 856 ± 74 μm^2^, representing an approximate 27% reduction (*p* < 0.01) in muscle fiber size as a result of extended exposure to microgravity (Fig. 2A, B). The average CSA of muscle fibers composing the quadriceps decreased from 1986 ± 330 μm^2^ to 1046 ± 201 μm^2^, representing an approximate 47% reduction (*p* < 0.01) in muscle fiber size (Fig. 2A, B). Therefore, the quadriceps displayed a greater magnitude of microgravity-induced atrophy (approximately 1.8× more based on fiber CSA) than the gastrocnemius.

Compared to ground controls, the overall abundance of slow-twitch fibers (those expressing MyHC I) in the gastrocnemius decreased from 18 to 5% (*p* < 0.01), with a reciprocal increase in the abundance of fast-twitch fibers (those expressing MyHC II) from 80 to 96% (*p* < 0.01) following 9 weeks of exposure to microgravity (Fig. 2A, C). This increase in fast-twitch fiber content occurred at the expense of existing slow-twitch fibers, as evidenced by co-labeled fibers (those expressing both MyHC I and II) in the gastrocnemius of spaceflown mice (Fig. 2D). By contrast, there was no evidence of a fiber type transition in the quadriceps as a consequence of its native 100% fast-twitch fiber type composition (Fig. 2A, C). These findings are consistent across images collected at high (Fig. 2A) and low (Fig. S2) magnifications.

### DGE and DAS are functionally distinct mechanisms of microgravity-induced transcriptome regulation

**Fig. 3:**
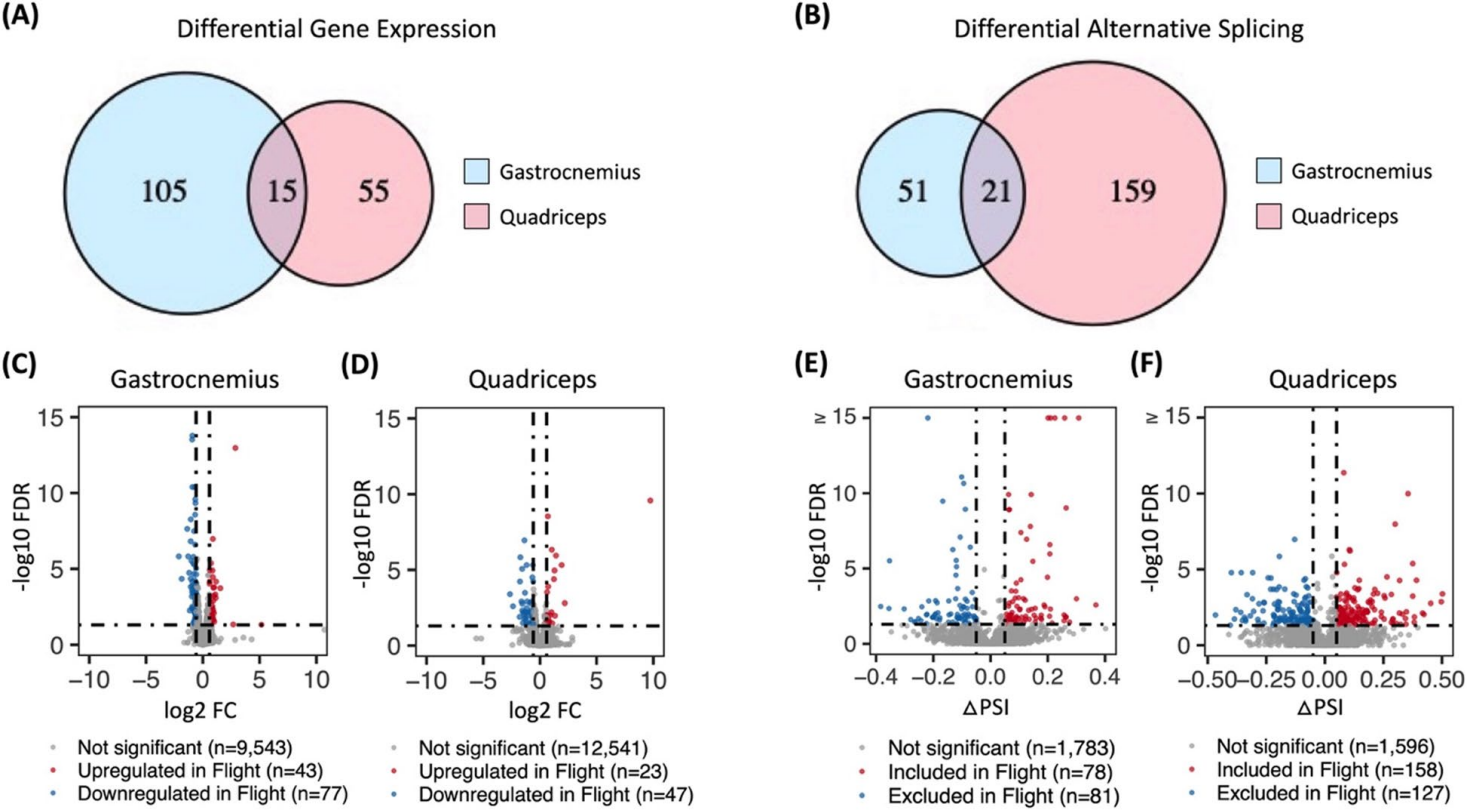
The microgravity-induced transcriptome is muscle type-specific. Venn diagrams compare the total number of **A** differentially expressed and **B** differentially alternatively spliced genes in the gastrocnemius (blue) and quadriceps (red). The size of each circle is representative of the number of genes that were differentially expressed or differentially alternatively spliced in each muscle. Numerical values are provided for genes unique to each muscle as well as those that were held in common between the two muscles, represented by overlapping regions. Volcano plots display significance (− log_10_ FDR) and fold change (log_2_ FC) of differentially gene expression across ground control and flight mice in the **C** gastrocnemius **D** and quadriceps, labeled for non-significant (grey), upregulated (red), and downregulated (blue) genes. Volcano plots display significance (− log10 FDR) and change in percent spliced in (△PSI) of differential alternative splicing across ground control and flight mice in the **E** gastrocnemius **F** and quadriceps, labeled for non-significant (grey), included (red), and excluded (blue) exon skipping or mutually exclusive exon events

In response to extended spaceflight, there was evidence of DGE (Additional files 1 and 2) and DAS (Additional files 3 and 4) in both the gastrocnemius and quadriceps. However, the microgravity-induced transcriptomes of these two muscles were distinct. Only approximately 8.5% of all DGE genes and approximately 9% of all genes with DAS events were held in common between the gastrocnemius and the quadriceps (Fig. 3A, B).

In the gastrocnemius, there were 120 DGE genes following 9 weeks of microgravity exposure, 43 of which were upregulated and 77 of which were downregulated (Fig. 3C). Upregulated genes displayed insignificant gene network enrichment, while downregulated genes were enriched for protein synthesis/processing, mitochondrial function, and, to a lesser extent, myosin heavy chain binding (Additional file 5). In the quadriceps, there were 70 DGE genes following 9 weeks of microgravity exposure, 23 of which were upregulated and 47 of which were downregulated (Fig. 3D). Upregulated genes displayed insignificant gene network enrichment, while downregulated genes were enriched for lipid metabolism (Additional file 5).

In the gastrocnemius, there were 159 DAS events in 72 genes following 9 weeks of microgravity exposure, 78 of which were included more in the flight group while 81 were excluded more in the flight group (Fig. 3E). Genes with either included or excluded DAS events were overwhelmingly found in structural and functional gene networks of skeletal muscle, including sarcoplasmic reticulum calcium ion transport, muscle contraction, and actin binding among others (Additional file 5). In the quadriceps, there were 285 DAS events in 180 genes following 9 weeks of microgravity exposure, 158 of which were included more in the flight group while 127 were excluded more in the flight group (Fig. 3F). Again, genes with DAS events were overwhelmingly enriched for structural and functional gene networks of skeletal muscle, including sarcomere organization, myofibril assembly, and muscle contraction among others (Additional file 5). Therefore, while there were approximately 1.75× more DAS events in the quadriceps as compared to the gastrocnemius, in both muscles, DAS, but not DGE, occurred in genes that encode proteins with known functions in skeletal muscle (referred to hereafter as skeletal muscle genes). The only exception to this finding was the DGE of eight skeletal muscle genes (*Actn2*, *Myl12a*, *Myl2*, *Myl3*, *Myom3*, *Myoz2*, *Tnnc1*, *Tnni1*) in the gastrocnemius of mice exposed to microgravity (Table 1). By contrast, in response to extended spaceflight, there were 32 potentially protein structure-altering DAS events in 15 skeletal muscle genes in the gastrocnemius (Table 2) and 68 potentially protein structure-altering DAS events in 25 skeletal muscle genes in the quadriceps (Table 3). Potentially protein structure-altering DAS events were defined as those which involve a region of the transcript that (i) encodes a functional domain of the resulting protein product, (ii) invokes a frameshift in the protein-coding sequence, or (iii) has been previously shown to alter the structure and/or function of the protein product in some other way.

**Table 1:**
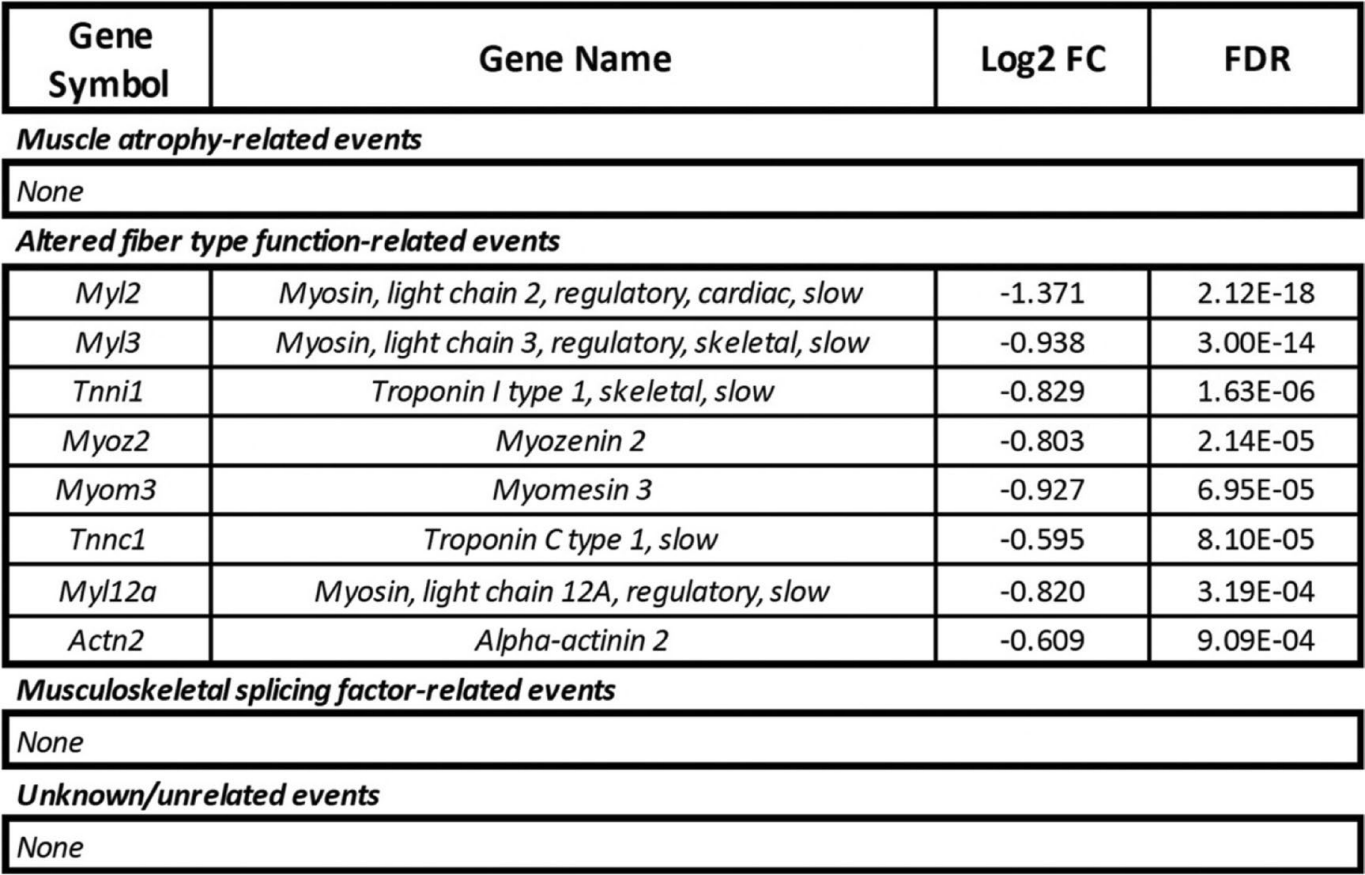
DGE of skeletal muscle genes in the gastrocnemius of spaceflown mice. Eight genes encoding proteins with known functions in skeletal muscle were differentially expressed in the gastrocnemius between flight and ground control groups. Genes were categorized into four groups based on previous research suggesting the expression of their encoded protein isoforms i) accompany muscle atrophy, ii) are associated with altered fiber type function, iii) are thought to compromise the function of musculoskeletal splicing regulators, or iv) have an unknown or unrelated perturbation. Genes were identified by gene symbol and gene name. Log_2_ fold change (log_2_ FC) values (positive values, greater expression in flight as compared to ground; negative values, less expression in flight as compared to ground) as well as FDR (false discovery rate-adjusted *p*-values) are provided for each gene. Genes are ordered by significance of DGE as measured by FDR

**Table 2:**
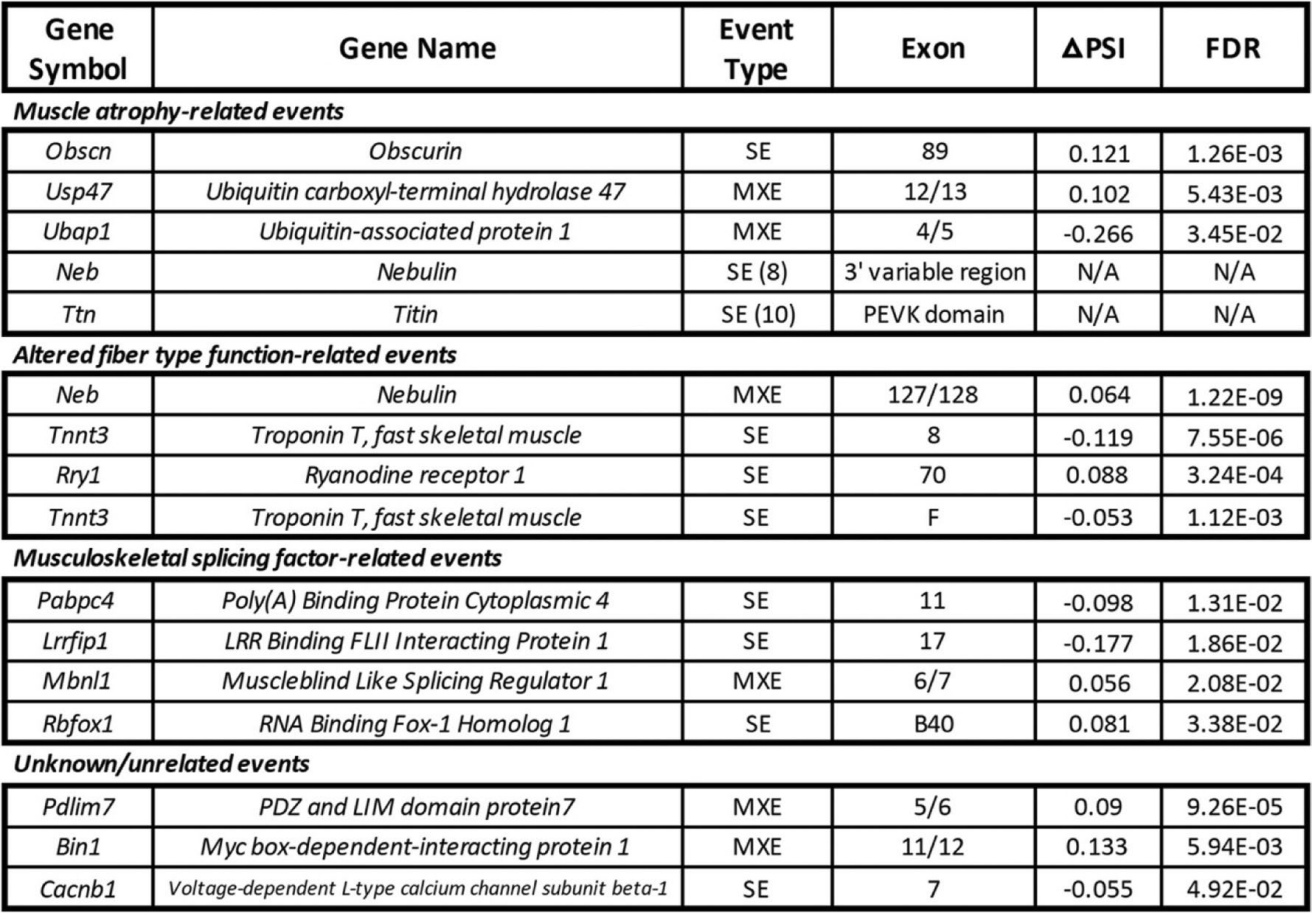
DAS of skeletal muscle genes in the gastrocnemius of spaceflown mice. Thirty-two significant DAS events were identified in 15 genes encoding proteins with known functions in skeletal muscle in the gastrocnemius between flight and ground control groups. DAS events were categorized into four groups based on previous research suggesting the expression of their encoded protein isoforms i) accompany muscle atrophy, ii) are associated with altered fiber type function, iii) are thought to compromise the function of musculoskeletal splicing regulators, or iv) have an unknown or unrelated perturbation. DAS events were identified by gene symbol, gene name, event type (SE, skipped exon; MXE, mutually exclusive exons), and involved exon(s). △PSI (change in percent spliced in of a specific exon) values (positive values, more inclusion in flight as compared to ground; negative values, less inclusion in flight as compared to ground) as well as FDR (false discovery rate-adjusted *p*-values) are provided for each DAS event. Parentheses with numerical values next to SE or MXE denotations [e.g., SE (8)] correspond to genes with multiple DAS events. For these genes, regions (e.g., 3′ variable region) rather than specific exons are provided. Genes are ordered by significance of DAS as measured by FDR

**Table 3:**
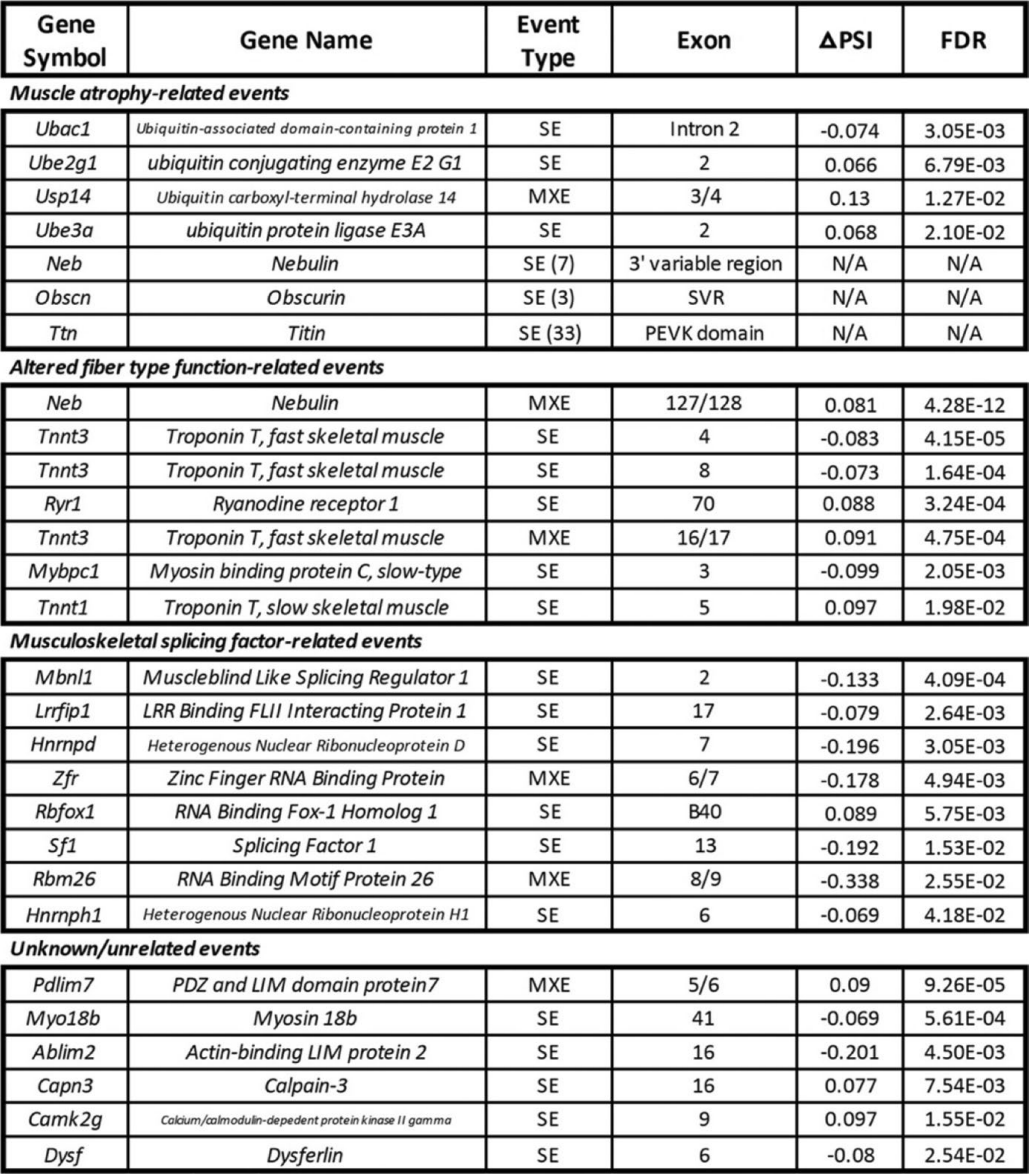
DAS of skeletal muscle genes in the quadriceps of spaceflown mice. Sixty-eight significant DAS events were identified in 25 genes encoding proteins with known functions in skeletal muscle in the quadriceps between flight and ground control groups. DAS events were categorized into four groups based on previous research suggesting the expression of their encoded protein isoforms i) accompany muscle atrophy, ii) are associated with altered fiber type function, iii) are thought to compromise the function of musculoskeletal splicing regulators, or iv) have an unknown or unrelated perturbation. DAS events were identified by gene symbol, gene name, event type (SE, skipped exon; MXE, mutually exclusive exons), and involved exon(s). △PSI (change in percent spliced in of a specific exon) values (positive values, more inclusion in flight as compared to ground; negative values, less inclusion in flight as compared to ground) as well as FDR (false discovery rate-adjusted *p*-values) are provided for each DAS event. Parentheses with numerical values next to SE or MXE denotations [e.g., SE (8)] correspond to genes with multiple DAS events. For these genes, regions (e.g., 3′ variable region) rather than specific exons are provided. Genes are ordered by significance of DAS as measured by FDR

**Fig. 4:**
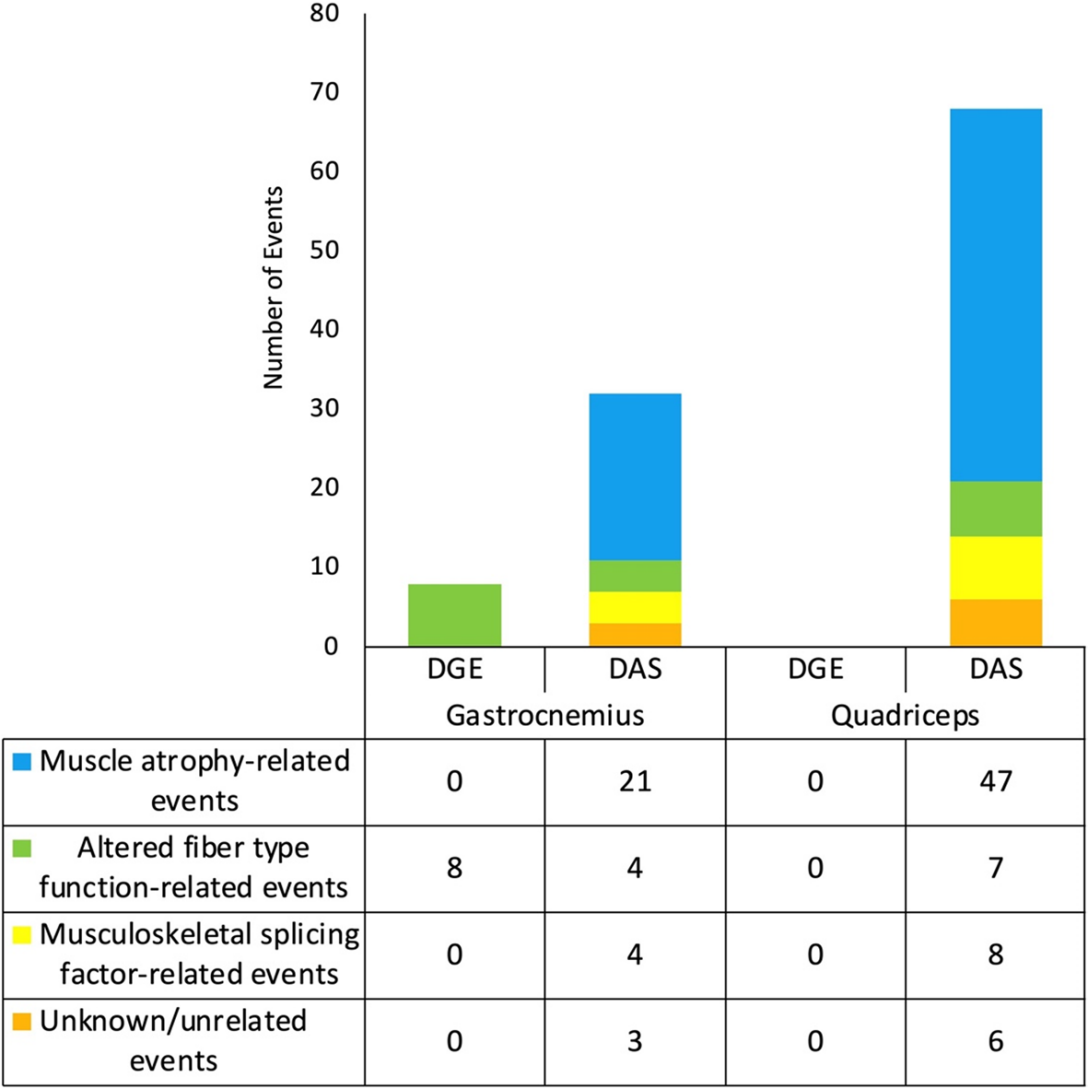
DAS, more than DGE, is associated with the chronic physiological adaptations of both the gastrocnemius and quadriceps to microgravity. Combined table and bar graphs depict the overall number of DGE and DAS events in genes encoding proteins with known functions in skeletal muscle. Events were categorized into four groups based on previous research suggesting the expression of their encoded protein isoforms (i) accompany muscle atrophy (blue), (ii) are associated with altered fiber type function (green), (iii) are thought to compromise the function of musculoskeletal splicing regulators (yellow), or (iv) have an unknown or unrelated perturbation (orange). The size of the colored sections of the bar graphs is representative of the number of events for each event category as shown in the table below. The cumulative height of the bar graphs is representative of the overall number of potentially functionally significant DGE or DAS events, regardless of category, in each muscle

Once we established that the skeletal muscle transcriptome undergoes extensive remodeling via DAS in spaceflight, we then sought to characterize each of these DAS events in more detail. Drawing from the literature, we organized Tables 1, 2 and 3 to display both DGE genes and genes with potentially protein structure-altering DAS events into four groups: (i) those known to accompany muscle atrophy; (ii) those associated with altered fiber type function; (iii) those thought to compromise the function of musculoskeletal splicing regulators; (iv) those with an unknown or unrelated perturbation. These findings are summarized in Fig. 4, where it can be seen that in both the gastrocnemius and quadriceps, chronic physiological adaptations of skeletal muscle to microgravity may be more reliant on DAS than DGE.

### Muscle atrophy is associated with microgravity-induced DAS of both the ubiquitin-proteasome pathway and transcripts encoding giant sarcomeric proteins

Protein degradation via the ubiquitin-proteasome pathway is a major cause of acute muscle atrophy, which represents muscle loss that occurs within approximately 48 h of an atrophy-inducing perturbation [57]. Acute activation of the ubiquitin-proteasome pathway has been shown to be directed by DGE [58]; however, a recent study in rats [39] discovered DAS of ubiquitin-proteasome pathway transcripts after 7 days of hindlimb unloading. Following 9 weeks of microgravity exposure, we observed DAS, but not DGE, of transcripts encoding ubiquitin-proteasome pathway proteins in both the gastrocnemius (two DAS events; see Table 2) and the quadriceps (four DAS events; see Table 3). Importantly, we identified potential gain-of-function splicing events within *Ubap1* and *Usp14* that serve as examples of possible chronic ubiquitin-proteasome pathway activation by DAS.

**Fig. 5:**
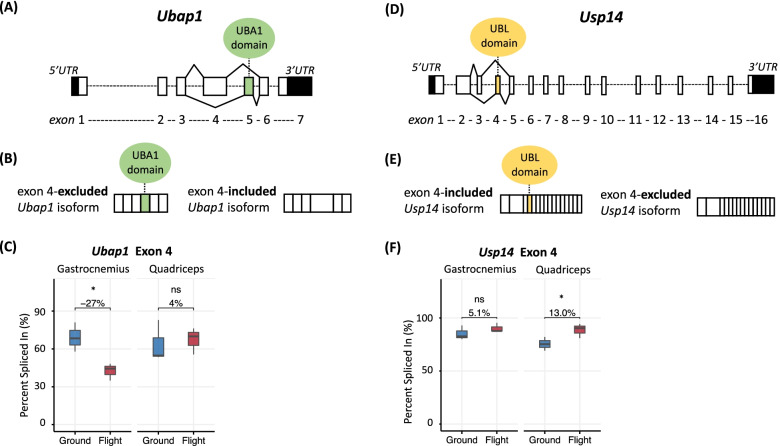
DAS of ubiquitin-proteasome pathway genes during spaceflight. **A** Bars and dashed lines represent exons and introns, respectively, of *Ubap1* pre-mRNA, with exon numbering below. Untranslated regions (UTR) are denoted in black. Solid lines connecting exons 4 and 5 to nearby exons represent mutually exclusive splicing. The UBA1 domain encoded by exon 5 is depicted in green. **B** The exon 4-excluded *Ubap1* splice isoform possesses the UBA1 domain-encoding region. The exon 4-included *Ubap1* splice isoform does not possess the UBA1 domain-encoding region. **C** Box-and-whisker plots depict the distribution (mean value, upper/lower quartiles, and maximum/minimum) of PSI (percent spliced in of a specific exon) values for exon 4 (first exon of mutually exclusive exon 4/5 event on + strand) in ground and flight for gastrocnemius (left pair) and quadriceps (right pair). △PSI (change in percent spliced in of a specific exon) values between ground and flight are provided in numerical form. Asterisks represent significance as reported by rMATS-turbo (^ns^*FDR* > 0.05, *FDR < 0.05). **D** Bars and dashed lines represent exons and introns, respectively, of *Usp14* pre-mRNA, with exon numbering below. Untranslated regions (UTR) are denoted in black. Solid lines connecting exons 3 and 4 to nearby exons represent mutually exclusive splicing. The UBL domain encoded by exon 4 is depicted in yellow. **E** The exon 4-included *Usp14* splice isoform possesses the UBL domain-encoding region. The exon 4-excluded *Usp14* splice isoform does not possess the UBL domain-encoding region. **F** Box-and-whisker plots depict the distribution (mean value, upper/lower quartiles, and maximum/minimum) of PSI (percent spliced in of a specific exon) values for exon 4 (first exon of mutually exclusive 3/4 event on − strand) in ground and flight for gastrocnemius (left pair) and quadriceps (right pair). △PSI (change in percent spliced in of a specific exon) values between ground and flight are provided in numerical form. Asterisks represent significance as reported by rMATS-turbo (^ns^*FDR* > 0.05, *FDR < 0.05)

*Ubap1*, which encodes ubiquitin-associated protein 1, undergoes DAS via mutual exclusion of exons 4 and 5 (Fig. 5A). Of most importance is the inclusion or exclusion of exon 5, which encodes a ubiquitin-associated (UBA) domain required for interaction of Ubap1 with ubiquitin [59]. The exon 4-excluded, exon 5-included transcript isoform encodes a functionally intact, UBA1-retained protein product while the exon 4-included, exon 5-excluded transcript isoform encodes a partially dysfunctional, UBA1-removed protein product (Fig. 5B). In the gastrocnemius of spaceflown mice, exon 4 was 27% less abundant following 9 weeks of microgravity exposure (FDR < 0.05; Fig. 5C), resulting in a reciprocal increase in exon 5 abundance. Therefore, the exon 4-excluded, exon 5-included, functionally intact, UBA1-retained protein product is likely expressed to a greater degree in spaceflight in the gastrocnemius. There was no significant difference in exon 4 or 5 inclusion across flight and ground control groups of the quadriceps (4%, FDR > 0.05; Fig. 5C).

*Usp14*, which encodes ubiquitin carboxyl-terminal hydrolase 14, undergoes DAS via mutual exclusion of exons 3 and 4 (Fig. 5D). Exon 4 encodes the ubiquitin-like (UBL) domain required for Usp14’s activation of ubiquitinated proteins and stimulation of the proteasome’s degradative capacity [60]. Therefore, the exon 3-excluded, exon 4-included transcript isoform encodes a functionally intact, UBL-retained protein product while the exon 3-included, exon 4-excluded transcript isoform encodes a partially dysfunctional, UBL-removed protein product (Fig. 5E). While there was no significant difference in exon 4 inclusion across flight and ground control groups of the gastrocnemius (5.1%, FDR > 0.05; Fig. 5F), exon 4 was 13% more abundant in the quadriceps of spaceflown mice as compared to ground controls (FDR < 0.05; Fig. 5F) at the reciprocal expense of exon 3. This indicates that the exon 3-excluded, exon 4-included, functionally intact, UBL-retained protein product is likely expressed to a greater degree in spaceflight in the quadriceps.

While AS events within a single transcript are often discussed in isolation of one another, numerous AS events may act concomitantly to impact the structure and function of long mRNA transcripts. In the context of skeletal muscle, concomitant AS of transcripts encoding the three giant sarcomeric proteins (*Ttn*, *Obscn*, and *Neb*) can invoke significant alternations to the size of the resulting protein products (titin, obscurin, and nebulin). Non-differentially expressed, *Ttn*, *Obscn*, and *Neb* were all differentially spliced following 9 weeks of spaceflight in both the gastrocnemius and quadriceps. Specifically, we observed significant alterations in the length of transcript regions encoding important functional domains that have been associated with muscle atrophy.

**Fig. 6:**
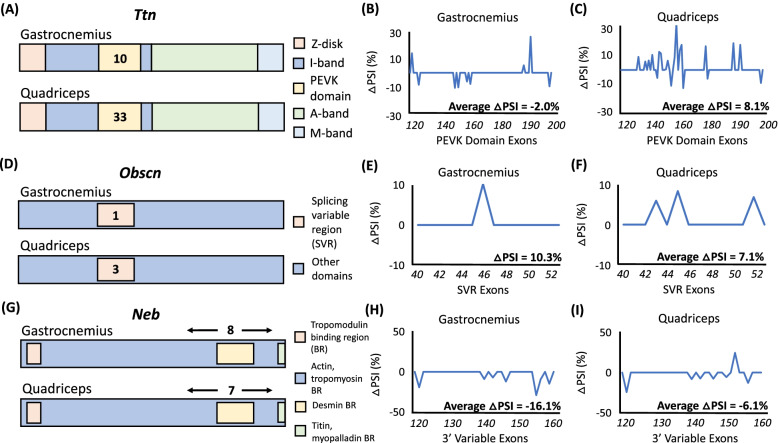
DAS of transcripts encoding giant sarcomeric proteins during spaceflight. **A** *Ttn,* **D** *Obscn,* and **G** *Neb* pre-mRNA transcript representations. Colored regions represent encoded protein domains described in the legends provided. Solid numbers represent the number of statistically significant DAS events within the corresponding region of interest in the gastrocnemius (top) and quadriceps (bottom). Line graphs display △PSI (change in percent spliced in of a specific exon) values for exons encoding the *Ttn* PEVK domain (exons 122-202), *Obscn* splicing variable region (SVR) (exons 40-52), and *Neb* C-terminal variable region (exons 120-160), respectively, that underwent significant DAS between ground control and flight groups in the gastrocnemius (**B**, **E**, **H**, respectively) and quadriceps (**C**, **F**, **I**, respectively). Positive △PSI events represent exons that were included more in the flight group while negative △PSI value events represent exons that were included less in the flight group. Non-significantly alternatively spliced exons were assigned a △PSI value of 0. Average △PSI across statistically significant events are provided

Titin, which is encoded by the gene *Ttn*, is a 3900-kDa protein that regulates the elasticity and contractile strength of the sarcomere via the length of its PEVK domain, named for its high proportion of Pro-Glu-Val-Lys amino acids. Often referred to as a “molecular spring”, lengthening of the PEVK domain has been associated with the development of muscle atrophy [61]. While there is a relatively equal distribution of positive and negative △PSI values (average △PSI across statistically significant events = − 2.0%; Fig. 6B) across the 10 significant DAS events within the PEVK domain-encoding region in the gastrocnemius (Fig. 6A), the majority of the 33 significant DAS events within the PEVK domain-encoding region in the quadriceps (Fig. 6A) exhibited a positive △PSI value (average △PSI across statistically significant events = 8.1%; Fig. 6C). The concomitant inclusion of alternatively spliced exons in the quadriceps is expected to lengthen titin’s PEVK domain during prolonged spaceflight in myofibers composing the quadriceps. This spaceflight-induced extension of the PEVK domain may contribute to the development of atrophy in the quadriceps, while a lack of extensive spaceflight-induced alterations to the PEVK domain in the gastrocnemius is expected considering its relatively lower level of atrophy as compared to the quadriceps in this study.

Obscurin, encoded by the gene *Obscn*, is an 800 kD protein that is integral to myofibril organization during assembly. DAS of the *Obscn* splicing variability region (SVR, exons 40–53) has been associated with muscle atrophy in rat models [38]. Specifically, exon inclusion within the SVR precipitates atrophic phenotypes. While the precise regulatory mechanism underlying the effect of DAS on *Obscn* during atrophy is yet to be fully elucidated, it is speculated that the inclusion of exons within the “GGGG”-rich SVR leads to the development of cytotoxic secondary RNA structures called G-quadruplexes [62]. In the gastrocnemius of mice exposed to microgravity for 9 weeks, there was evidence of one exon within the *Obscn* SVR that was included to a significantly higher degree in microgravity (Fig. 6D, E); however, in the quadriceps of mice exposed to microgravity for 9 weeks, we identified three exons within the *Obscn* SVR that were included to a significantly higher degree in microgravity compared to ground controls (Fig. 6D, F). This is similar in magnitude to the four-exon inclusion identified in the atrophic rat model employed by Qiu et al. [38]. Exon inclusion within the *Obscn* SVR during prolonged spaceflight is expected to increase the prevalence of cytotoxic G-quadruplexes in myofibers composing both the gastrocnemius and quadriceps, which may be contributing to the development of atrophy in both muscles, albeit to a lesser degree in the gastrocnemius than in the quadriceps in this study.

Nebulin is a 600–900-kD protein encoded by the gene *Neb* that determines thin filament length. In contrast to titin, shortening of nebulin has been associated with the development of muscle atrophy [63]. In both the gastrocnemius and quadriceps of mice exposed to microgravity for 9 weeks, there was DAS (eight and seven significant DAS events, respectively) within the 3′ region of the *Neb* transcript; involved 3′ exons encode the actin, tropomyosin, and desmin binding regions of nebulin (Fig. 6G). The average statistically significant △PSI values across all DAS events were − 16.1% and − 6.1% in the gastrocnemius and quadriceps, respectively (Fig. 6H, I). The concomitant exclusion of alternatively spliced exons in the gastrocnemius and quadriceps during prolonged spaceflight is expected to shorten nebulin’s C-terminal variable region in myofibers composing both the gastrocnemius and quadriceps. This spaceflight-induced shortening of nebulin may contribute to the development of atrophy in both the gastrocnemius and quadriceps.

### DGE is associated with reduction of slow-twitch fiber content while DAS is associated with potentially expanded fast-twitch fiber function

Reliance on the dynamic component of motor function in microgravity necessitated an adaptive shift towards a greater overall fast-twitch fiber phenotype in the hindlimb muscles of mice exposed to microgravity for 9 weeks. The gastrocnemius, which expressed both slow- and fast-twitch muscle fibers prior to spaceflight, underwent a fiber type transition in which fast-twitch fiber content increased significantly at the expense of native slow-twitch fibers (see Fig. 2). By comparison, the quadriceps maintained its 100% fast-twitch dominance (see Fig. 2). Considering the gastrocnemius underwent a fiber type transition while the quadriceps exhibited fiber type maintenance following 9 weeks of microgravity exposure, we investigated whether these differences in the microgravity-induced fiber type alterations of the gastrocnemius and quadriceps were accompanied by differences in DGE and DAS of fiber type-related genes during spaceflight in these two muscles.

First, we found that all differentially expressed fiber type-related genes in the gastrocnemius (*Actn2*, *Myl12a*, *Myl2*, *Myl3*, *Myom3*, *Myoz2*, *Tnnc1*, *Tnni1*; see Table 1) encoded slow-twitch-specific proteins [64], and all such transcripts were downregulated following extended spaceflight. By contrast, in the quadriceps of mice exposed to microgravity there were no differentially expressed fiber type-related genes (see Additional file 2). Therefore, the diminished slow-twitch fiber content in the gastrocnemius that is not observed in the quadriceps may be explained by the downregulation of slow twitch-specific transcripts in the gastrocnemius but not the quadriceps.

While no fiber type-related genes were differentially expressed in the quadriceps during prolonged exposure to microgravity, there were five fiber type-related genes (*Tnnt1*, *Mybpc1*, *Tnnt3*, *Neb*, and *Ryr1*; see Table 3) that underwent potentially protein structure-altering DAS events, three of which (*Neb*, *Ryr1*, and *Tnnt3*; see Table 2) were also observed in the gastrocnemius of mice exposed to microgravity. Considering the quadriceps exhibited no change in fiber type composition after 9 weeks of microgravity exposure, these DAS events were investigated in more detail for their potential to impact the function of native fast-twitch fibers.

**Fig. 7:**
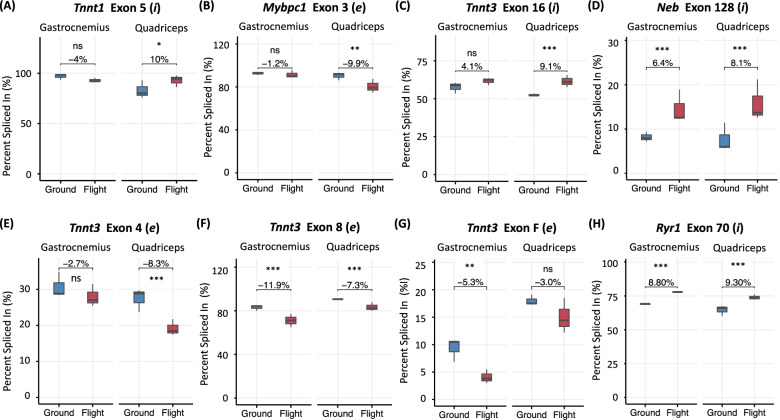
DAS of fiber type-specific genes during spaceflight. Box-and-whisker plots depict the distribution (mean value, upper/lower quartiles, and maximum/minimum) of PSI (percent spliced in of a specific exon) values of **A** *Tnnt1* exon 5, **B** *Mybpc1* exon 3, **C** *Tnnt3* exon 16, **D** *Neb* exon 128, **E** *Tnnt3* exon 4, **F** *Tnnt3* exon 8, **G** *Tnnt3* exon F, and **H** *Ryr1* exon 70 in ground and flight for gastrocnemius (left pair) and quadriceps (right pair). Beside each gene name and involved exon is an (*i*) or (*e*), which represents the typical AS pattern (*i*, included; *e*, excluded) for that exon in fast-twitch fibers. For mutually exclusive exon events (e.g., *Tnnt3* exons 16 and 17) the PSI value indicates the percent inclusion of the exon listed first in the pair based on whether the transcript is on the + or − strand. △PSI (change in percent spliced in of a specific exon) values between ground and flight are provided in numerical form. Asterisks represent significance as reported by rMATS-turbo (^ns^*FDR* > 0.05, *FDR < 0.05, **FDR < 0.01, ***FDR < 0.001)

For example, *Tnnt1* and *Mybpc1* encode the slow-twitch isoforms of troponin T (Tnnt) and myosin binding protein-C (MyBP-C), respectively; however, these canonical slow-twitch transcripts can be alternatively spliced such that the resulting protein isoforms mirror the function of their fast-twitch counterparts. Specifically, exon 5-included *Tnnt1* and exon 3-excluded *Mybpc1* transcripts have been abundantly observed in fast-twitch muscle fibers. In the case of *Tnnt1*, inclusion or exclusion of exon 5 alters the three-dimensional structure of Tnnt and subsequently influences the calcium (Ca^2+^) sensitivity of the troponin complex [65, 66]. The predicted superior Ca^2+^ sensitivity of the exon 5-included compared to the exon 5-excluded Tnnt isoform contributes to the preferential inclusion of exon 5 in *Tnnt1* transcripts within fast-twitch muscle fibers [67, 68]. As for *Mybpc1*, exon 3 of *Mybpc1* falls within the region encoding the actin and myosin binding regions of the resulting protein product and modulates actin-myosin binding and sliding in a variant-specific manner [69]. Exclusion of exon 3 has been abundantly observed in *Mybpc1* transcripts expressed in fast-twitch muscle, with resulting changes in MyBP-C protein phosphorylation having been proposed to facilitate enhanced actomyosin cross-bridge formation [70, 71]. Therefore, increased abundance of *Tnnt1* exon 5 (10%, FDR < 0.05; Fig. 7A) and decreased abundance of *Mybpc1* exon 3 (− 9.9%, FDR < 0.01; Fig. 7B) in the quadriceps of mice exposed to microgravity are both potentially functionally significant DAS events associated with expansion of fast-twitch fiber function during prolonged spaceflight.

Other altered fiber type function-related DAS events annotated following 9 weeks of microgravity exposure include the mutual exclusion of *Tnnt3* exons 16/17 and *Neb* exons 127/128. While *Tnnt1* encodes the canonical slow-twitch isoform of troponin T, *Tnnt3* encodes its fast-twitch isoform. The fast-twitch function of Tnnt is regulated by DAS within the 3′ region of *Tnnt3* that encodes the C-terminal binding domains for troponin I and tropomyosin [72]. Specifically, exon 16 and exon 17 of *Tnnt3* vary in their sequence similarity to the functionally equivalent exon in *Tnnt1*; exon 17 shows a much higher degree of similarity (61%) than exon 16 (32%) (Wang and Jin, [73]). As a consequence, the binding affinity of Tnnt for its functional partners (troponin I and tropomyosin) is variable [74]; the affinity of the exon 17-included Tnnt isoform is higher for the slow-twitch isoforms of troponin I and tropomyosin, whereas the affinity of the exon 16-included Tnnt isoform is higher for the fast-twitch isoforms of troponin I and tropomyosin (Wang and Jin, [73]). While there are no slow- or fast-twitch gene isoforms of *Neb*, the alternatively spliced isoforms of *Neb* have displayed fiber type specificity. For instance, the exon 128-included and exon 127-included isoforms are more abundant in fast-twitch and slow-twitch dominant muscle types, respectively [75–77]. Therefore, increased abundance of *Tnnt3* exon 16 (9.1%, FDR < 0.001; Fig. 7C) at the reciprocal expense of *Tnnt3* exon 17, along with the increased abundance of *Neb* exon 128 (8.1%, FDR < 0.001; Fig. 7D) at the reciprocal expense of *Neb* exon 127 in the quadriceps of mice exposed to microgravity provides additional evidence of the possible expansion of fast-twitch fiber function via DAS during prolonged spaceflight. The impact of DAS was also observed in the gastrocnemius, as evidenced by the increased abundance of *Neb* exon 128 (6.4%, FDR < 0.001; Fig. 7D) at the reciprocal expense of *Neb* exon 127 in the gastrocnemius of mice exposed to microgravity.

**Fig. 8:**
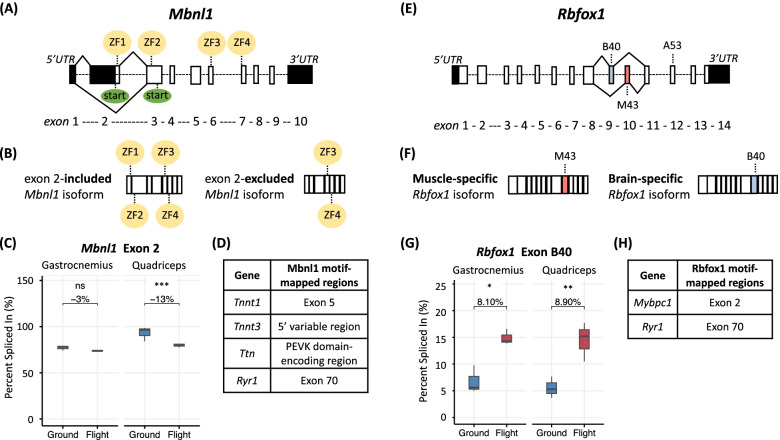
DAS of musculoskeletal splicing regulators during spaceflight. **A** Bars and dashed lines represent exons and introns, respectively, of *Mbnl1* pre-mRNA with exon numbering below. Untranslated regions (UTR) are denoted in black. Solid lines connecting exon 2 to nearby exons represent exon skipping. Two encoded start codons are depicted in green. Four encoded zinc-finger domains (ZF1-4) are depicted in yellow. **B** The exon 2-included *Mbnl1* splice isoform possesses ZF1, 2, 3, and 4-encoding regions. The exon 2-excluded *Mbnl1* splice isoform possesses only ZF3 and 4-encoding regions. **C** Box-and-whisker plots depict the distribution (mean value, upper/lower quartiles, and maximum/minimum) of PSI (percent spliced in of a specific exon) values for exon 2 in ground and flight for gastrocnemius (left pair) and quadriceps (right pair). △PSI (change in percent spliced in of a specific exon) values between ground and flight are provided in numerical form. Asterisks represent significance as reported by rMATS-turbo (^ns^*FDR* > 0.05, *FDR < 0.05, **FDR < 0.01, ***FDR < 0.001). **D** Table depicts genes with known Mbnl1 binding motifs. **E** Bars and dashed lines represent exons and introns, respectively, of *Rbfox1* pre-mRNA with exon numbering below. Untranslated regions (UTR) are denoted in black. Solid lines connecting exons B40 (light blue) and exon M43 (red) to nearby exons represent mutually exclusive splicing. **F** The brain-specific *Rbfox1* splice isoform has exon B40 included and exon M43 excluded, while the muscle-specific *Rbfox1* splice isoform has exon B40 excluded and exon M43 included. **G** Box-and-whisker plots depict the distribution (mean value, upper/lower quartiles, and maximum/minimum) of PSI (percent spliced in of a specific exon) values for exon B40 in ground and flight for gastrocnemius (left pairs) and quadriceps (right pairs). △PSI (change in percent spliced in of a specific exon) values between ground and flight are provided in numerical form. Asterisks represent significance as reported by rMATS-turbo (^ns^*FDR* > 0.05, *FDR < 0.05, **FDR < 0.01, ***FDR < 0.001). **H** Table depicts genes with known Rbfox1-binding motifs

Similar to *Neb*, there were two other genes (*Tnnt3*, *Ryr1*) with altered fiber type function-related DAS events identified in the gastrocnemius of mice exposed to microgravity, both of which were held in common with the quadriceps of spaceflown mice. In addition to regulation via splicing within its 3′ region, *Tnnt3* also undergoes extensive splicing within its 5′ variable region. While the N-terminal variable region of Tnnt has no known binding partners in the thin filament regulatory system, alternative splicing within the 5′ variable region of *Tnnt3* generates various protein isoforms that fall into either acidic residue-enriched or basic residue-enriched isoform classes [78]. Changes in the charge of the N-terminal variable region alter the three-dimensional structure of the resulting protein and influence the Ca^2+^ sensitivity of the troponin complex. Specifically, basic isoforms, which exclude exons 4, 8, and F, tend to invoke a greater Ca^2+^ sensitivity to contraction [65, 78], contributing to their preferential utilization in fast-twitch skeletal muscle fibers [67, 68]. In the gastrocnemius, while there was no significant DAS of exon 4 (− 2.7%, FDR > 0.05, Fig. 7E), there was significant exclusion of exons 8 (− 11.9%, FDR < 0.001, Fig. 7F) and F (− 5.3%, FDR < 0.01; Fig. 7G). Exons 4 (− 8.3%, FDR < 0.001; Fig. 7E) and 8 (− 7.3%, FDR < 0.001; Fig. 7F) were significantly excluded in the quadriceps, while exon F was not significantly alternatively spliced (− 3.0%, FDR > 0.05, Fig. 7G). Although exhibiting partially distinct splicing patterns in the gastrocnemius and quadriceps, both muscle types displayed evidence of DAS within the 5′ variable region of *Tnnt3* that would promote the expression of basic residue-enriched protein isoforms, presumably to cope with the increased Ca^2+^ demand in fast-twitch fibers during prolonged spaceflight. In addition, there is evidence to suggest that DAS of *Ryr1* may contribute to the function of fast-twitch muscles. Specifically, the human homolog of *Ryr1* exon 70 (*RYR1* exon 70) is known to be preferentially included in the spliced mRNA of fast-twitch muscles and reciprocally excluded in slow-twitch muscles [79]. While the mechanism that contributes to increased use of this isoform in fast-twitch muscles remains unknown, the microgravity-induced inclusion of exon 70 in *Ryr1* in both the gastrocnemius (8.8%, FDR < 0.001; Fig. 7H) and quadriceps (9.3%, FDR < 0.001; Fig. 7H) can be taken as further evidence of the possible splicing-supported expansion of fast-twitch fiber function during prolonged spaceflight in both muscle types.

### DAS of musculoskeletal splicing regulators may account for downstream splicing changes in spaceflight

DAS is under the regulatory control of RNA-binding proteins (RBPs), with DGE of these RBPs thought to invoke downstream changes in DAS [23]. However, despite identifying numerous spaceflight-induced DAS events, there was no evidence of spaceflight-induced DGE of RBPs (see Additional files 1 and 2). Surprisingly, we found evidence of potentially protein structure-altering DAS of RBPs themselves; in the gastrocnemius, there were four significant DAS events in four RBP-encoding transcripts as compared to eight significant DAS events in eight RBP-encoding transcripts in the quadriceps (see Tables 2 and 3). Of specific interest are *Mbnl1* and *Rbfox1*, because of reports that Mbnl1 directs splicing of *Tnnt1*, *Tnnt3*, *Ttn*, and *Ryr1* [80] and Rbfox1 directs splicing of *Mybpc1* and *Ryr1* [81].

Mbnl1 is an RBP with two tandem *trans*-acting RNA-binding domains [zinc finger (ZF)1-2 tandem and ZF3-4 tandem] that bind *cis-*regulatory elements in mRNA targets, including *Tnnt1*, *Tnnt3*, *Ttn*, and *Ryr1* (Fig. 8D), and direct splicing of nearby transcript regions [80]. *Mbnl1* undergoes DAS of exon 2 with the generation of two isoforms, each with a different translational start codon (Fig. 8A). In the exon 2-included transcript isoform, the start codon resides in exon 2 and the final protein isoform contains both tandem RNA-binding domains (ZF1-2 and ZF3-4). However, in the exon 2-excluded transcript isoform, the start codon resides in exon 3 and the final protein isoform contains only one of two tandem RNA-binding domains (ZF3-4, Fig. 8B). The ZF1-2 and ZF3-4-containing protein isoform exhibits more activity and target RNA motif specificity than the protein isoform containing ZF3-4 alone [82]. In the gastrocnemius of spaceflown mice, there was no significant change in exon 2 inclusion (− 3.0%, FDR > 0.05; Fig. 8C), but in the quadriceps of mice exposed to microgravity, the exon 2-included transcript isoform, which encodes both the ZF1-2 and ZF 3-4 tandem domains, is 13% less abundant (FDR < 0.001; Fig. 8C). So, while the activity and binding specificity of Mbnl1 likely remains intact in the gastrocnemius of spaceflown mice, Mbnl1 is expected to be expressed in a functionally impaired form in the quadriceps of mice exposed to microgravity.

**Fig. 9:**
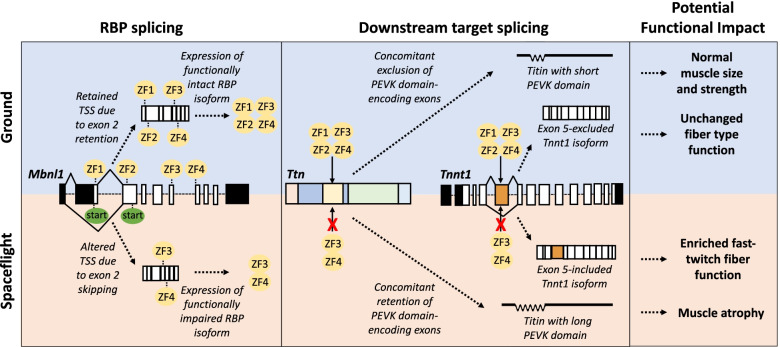
Schematic representation of physiological significant DAS of skeletal muscle genes via upstream splicing of RBPs. RBP splicing, downstream target splicing, and potential functional impact are highlighted here. Splicing patterns in ground and spaceflight are differentiated by blue and tan/orange boxes, respectively. Dotted lines represent directionality of proposed processes with corresponding text annotations. Solid lines represent RBP binding and splicing regulation with inhibition of such depicted by a red “X”. DAS of exon 2 of *Mbnl1* determines the translational start site (TSS) of *Mbnl1* mRNA and generates two distinct Mbnl1 RBP isoforms (ground, functionally intact; spaceflight, functionally impaired). While the functionally intact Mbnl1 isoform in ground binds freely to motif mapped regions of *Ttn* (PEVK domain-encoding exons) and *Tnnt1* (exon 5), the functionally impaired Mbnl1 in spaceflight exhibits less activity and target RNA motif specificity, directing splicing away from the canonical splicing pathway (*Ttn*, concomitant exclusion of PEVK domain-encoding exons; *Tnnt1*, exon 5 exclusion) and towards an aberrant splicing pathway (*Ttn*, concomitant inclusion of PEVK domain-encoding exons; *Tnnt1*, exon 5 inclusion) that has been associated with the physiological adaptations of skeletal muscle to spaceflight, such as expanded fast-twitch fiber function and muscle atrophy

The aberrant splicing of *Mbnl1* in the quadriceps is speculated to account for some of the splicing changes we identified in its downstream targets. For example, Mbnl1 has been motif mapped to the PEVK domain-encoding region of *Ttn* and exon 5 of *Tnnt1*. Homozygous knockout of *Mbnl1* results in increased inclusion of *Ttn* PEVK-encoding exons and *Tnnt1* exon 5, suggesting that Mbnl1 acts canonically to promote skipping of these exons [80]. This is consistent with our annotation of increased inclusion of *Ttn* PEVK-encoding exons (see Fig. 6) and *Tnnt1* exon 5 (see Fig. 7) in the spaceflown quadriceps, which increasingly expresses the exon 2-excluded, potentially dysfunctional protein-encoding transcript isoform of *Mbnl1*. In addition, the lack of DAS of *Ttn* PEVK-encoding exons (see Fig. 6) and *Tnnt1* exon 5 (see Fig. 7) in the spaceflown gastrocnemius is consistent with the expression of the exon 2-included, functional protein-encoding *Mbnl1* transcript isoform in this muscle. This proposed mechanism of physiologically significant DAS of skeletal muscle target genes via upstream splicing of RBP transcripts is outlined in Fig. 9 using Mbnl1and its downstream targets, *Ttn* and *Tnnt1*, as an example.

In addition, Rbfox1 is an RBP that acts as a splicing regulator in both muscle and brain tissue, with the tissue-specific function of this protein being regulated by DAS involving B40 (the brain-specific exon consisting of 40 base pairs) and M43 (the muscle-specific exon consisting of 43 base pairs) (Fig. 8E, F) [83]. In muscle, Rbfox1 is known to regulate splicing of *Mybpc1* and *Ryr1* (Fig. 8H) [81]. While the B40/M43 DAS event has previously been annotated as a mutually exclusive event, we observed significantly increased inclusion of B40 in both the gastrocnemius (8.1%; FDR < 0.05; Fig. 8G) and quadriceps (8.9%; FDR < 0.01; Fig. 8G), albeit without a reciprocal significant exclusion of M43 in either muscle. The preferential inclusion of B40 in *Rbfox1* transcripts in both the quadriceps and gastrocnemius may result in increasingly impaired binding of this RBP to its pre-mRNA targets in both muscles.

Similar to *Mbnl1*, the aberrant splicing of *Rbfox1* may account for some of the splicing changes we identified in its downstream targets. For example, Rbfox1 has been motif mapped to exon 3 of *Mybpc1*. Homozygous knockout of *Rbfox1* results in increased exclusion of *Mybpc1* exon 3 [81]. This is consistent with our annotation of significant exon 3 exclusion in the quadriceps, which undergoes splicing of *Rbfox1* to potentially functionally inhibitory isoforms. Despite potentially inhibitory splicing of *Rbfox1* in the gastrocnemius, we did not identify DAS of *Mybpc1* at exon 3 in this muscle, possibly due to the smaller magnitude of B40 inclusion in the gastrocnemius as compared to the quadriceps.

## Discussion

We set out to characterize the role of DAS in the transcriptomic response of mouse hind limb muscles to microgravity; however, the work presented here has also provided novel insights more broadly into the reciprocal relationship between DGE and DAS. Co-transcriptional splicing was first documented as long as 30 years ago [84], and since then, the molecular mechanisms underlying the coupling of DGE and DAS have been elucidated, including regulation of splicing by transcriptional elongation rate [85] and modulation of splicing factor recruitment by nucleosome positioning [86] and histone modifications [87]. In addition, more recent research has characterized a direct physical connection between RNA polymerase II and the spliceosome at the point of emergence of pre-mRNA from the transcriptional machinery [88]. Together, this evidence suggests that these distinct mechanisms of transcriptome regulation (transcription and splicing) are intricately related. While it is believed that this intricate relationship is regulated by tissue-specific regulatory factors, DGE and DAS have either been investigated together in a single biological system [89, 90] or separately across multiple biological systems [91, 92]. Therefore, there have been no comprehensive investigations of DGE and DAS together across multiple biological systems until now. Our results showed that the transcriptomes of the gastrocnemius and quadriceps were biased towards adaptation via DGE and DAS, respectively, after 9 weeks of microgravity exposure (see Fig. 3). This observation is the first evidence of a possible reciprocal, muscle type-specific relationship between DGE and DAS, such that each muscle preferentially employs one mechanism of transcriptome regulation at the other mechanism’s expense.

We hypothesize that the muscle type-specific regulatory biases we identified are indicative of differences in energy availability across each muscle. While it has been proposed that energy availability influences patterns of transcriptome regulation, only recently has it been shown that patterns of both DGE and DAS vary across high and low energy environments [93] as a result of diminished supply of mitochondrial adenine nucleotides in atrophic skeletal muscle [94, 95]. Potentially diminished adenine nucleotide supply in the more atrophied quadriceps may direct the use of DAS, a less energy-dependent mode of transcriptome regulation, over DGE, a more energy-dependent mode of transcriptome regulation [96, 97]. By contrast, potentially adequate supply of adenine nucleotides in the less atrophied gastrocnemius may favor DGE over DAS. To examine this possibility, further studies of microgravity-induced muscle atrophy should compare adenine nucleotide levels across various skeletal muscle types.

Regardless of the mechanism of transcriptome regulation that was preferentially employed in each muscle during prolonged spaceflight, we found that transcripts encoding proteins with known functions in skeletal muscle were primarily alternatively spliced rather than differentially expressed in both the gastrocnemius and quadriceps after 9 weeks of microgravity exposure (see Fig. 4). Therefore, while we identified regulation of mitochondrial function and lipid metabolism via DGE (see Additional file 5), as has been described previously in skeletal muscle following spaceflight [19–21], we characterized DAS as a novel means of modifying the transcriptome in response to microgravity. Further, we identified coordinate changes in splicing and microgravity-induced physiological adaptations of hind limb muscle, which included muscle atrophy (see Figs. 5 and 6) and potential expansion of fast-twitch function (see Fig. 7). Finally, in the absence of significant DGE of RBPs in either of the hind limb muscles studied here following microgravity exposure for 9 weeks, we discovered potentially functionally significant, spaceflight-induced DAS of RBPs themselves (see Fig. 8), an upstream provocation that may account for downstream splicing changes we identified in skeletal muscle transcript targets. Together, these findings represent the first parallel observations of splice variants and physiological adaptations of skeletal muscle to microgravity. These findings are supported by the extensive body of cited literature from models of microgravity as well as non-microgravity contexts that either annotated similar splicing-phenotype associations or established causative splicing-phenotype relationships by perturbing isoform-specific expression and characterizing downstream changes in muscle physiology. Ultimately, our work adds to the growing body of research demonstrating the power and potential of alternative splicing to affect skeletal muscle physiology and creates a resource for future mechanistic investigations of alternatively spliced skeletal muscle gene products and physiological adaptations of skeletal muscle to microgravity.

Beyond the associative, not necessarily causative nature of our work, we are also aware of four limitations of our study related to its design, including the age, gender, and strain of the mice employed and the timeline of our experimentation. First, previous characterizations of the musculoskeletal adaptation to spaceflight have occurred in mice ranging from 8-20 weeks old at the time of spaceflight [19, 20, 98, 99], requiring inquiry as to the role age (our experiments employed mice that were 30 weeks old at the beginning of experimentation) may have played in generating the muscle phenotypes we observed and the transcriptomic alterations we characterized. Although our mice are relatively older than those employed in previous microgravity investigations, the physiological adaptations of skeletal muscle we identified are consistent with those characterized previously in younger mice [19, 98, 99]. This is expected considering that sarcopenia typically does not present until at least two years of age in mice living in normal gravity [100]. More important to our work is the relationship between modes of transcriptome regulation and age. While relative employment of DGE and DAS fluctuates with age and organismal development, most of this fluctuation in mice occurs immediately preceding and immediately following birth (until approximately post-natal day 28) [89]. In addition, there is evidence in both mice and humans to suggest relative stability of the skeletal muscle transcriptome across adult years and even into later life [101]. Therefore, it is less likely that the transcriptomic alterations we characterized are simply a product of the age of the mice employed. In fact, investigations of microgravity-induced differential gene expression in younger mice [19, 20] showed downregulation of metabolic and mitochondrial pathways, similar to what we identified in our older mice. Therefore, while DAS has not been adequately investigated in the context of spaceflight, it can be speculated that extensive microgravity-induced DAS may be identified regardless of age. Despite this, future studies investigating microgravity-induced DAS across mice of varying ages would provide vital context for evaluating age-dependent transcriptomic responses to prolonged spaceflight.

Second, previous characterizations of the transcriptomic and physiologic adaptations of skeletal to spaceflight have occurred in female [98], male [20], and both female and male [99] mice, requiring inquiry as to the role gender (our experiments employed only female mice) may have played in generating the phenotypes we observed and the transcriptomic alterations we characterized. There is evidence in mice of sexual dimorphism in muscle physiology. Specifically, female mice tend to have greater type I (slow-twitch) fiber content [102] and smaller fiber CSA [103] than male mice. As a result of these anatomical differences, female mice tend to be more susceptible to both slow-to-fast fiber type alterations and muscle atrophy during hindlimb unloading [104]. Therefore, the magnitude of the microgravity-induced phenotypes we observed were likely magnified compared to what would be expected in male mice. More important to our work is the relationship between modes of transcriptome regulation and biological sex. In mouse models, sexually dimorphic patterns of alternative splicing have only been analyzed during sex determination in utero [105]. In humans, a comprehensive skeletal muscle transcriptome comparison between males and females revealed significant sex differences in the skeletal muscle transcriptome both at the level of DGE and DAS. Female-enriched transcript isoforms were associated with mitochondrial function, metabolism of acids and ketones, oxidation and reduction, cellular respiration, and fatty acid metabolism. By contrast, male-enriched transcript isoforms were found in the cytoplasm and proteasome, and enriched biological processes were almost all related to protein catabolism [106]. Ultimately, the sexual dimorphism of the skeletal muscle transcriptome suggests that while DAS is likely to be identified in both female and male mice exposed to microgravity, the downstream skeletal muscle gene targets may be sex-specific. Future studies investigating microgravity-induced DAS in male mice would provide vital context for evaluating sex differences in the transcriptomic response to prolonged spaceflight.

Third, many of the previous characterizations of the musculoskeletal adaptation to spaceflight have occurred in C57BL/6 mice [19, 98, 99], requiring inquiry as to the role strain (our experiments employed BALB/c mice) may have played in generating the muscle phenotypes we observed and the transcriptomic alterations we characterized. Similar to the discussion of the relatively advanced age of our mice, the physiological adaptations of skeletal muscle we identified are consistent with those characterized previously in C57BL/6 mice [19, 98, 99] despite evidence to suggest strain differences in muscular remodeling, albeit in a non-microgravity context [107]. More important to our work is the relationship between modes of transcriptome regulation and mouse strain. Of note, a recent multi-omics analysis of data from NASA’s GeneLab [108] revealed that C57BL/6 mice were more responsive at a transcriptomic level to spaceflight than BALB/c mice, as evidenced by 5–10× more differentially expressed genes on average in C57BL/6 datasets than BALB/c datasets. Although Beheshti et al. [108]’s work was limited to transcriptomic investigations of the liver, their findings are consistent with the approximately 10× less differentially expressed genes that we identified in the BALB/c quadriceps following exposure to microgravity (70 DGE genes; see Fig. 3) as compared to what Chakraborty et al. [20] identified in the C57BL/6 quadriceps following exposure to microgravity (776 DGE genes). Although these strain-specific differences in DGE have yet to be investigated in the context of DAS, available evidence indicates that the transcriptomic adaptation of BALB/c mice may be less profound compared to C57BL/6 mice. Future studies investigating microgravity-induced DAS across C57BL/6 and BALB/c mice would provide vital context for evaluating strain differences in the comprehensive transcriptomic response to prolonged spaceflight.

Fourth, previous characterizations of the musculoskeletal adaptation to spaceflight have typically occurred following 2–4 weeks of exposure to microgravity [19, 20, 98], requiring inquiry as to the role time (our samples were collected following 9 weeks of microgravity exposure) may have played in generating the muscle phenotypes we observed and the transcriptomic alterations we characterized. Cadena et al. [109] found that muscle atrophy is dynamic, with atrophy peaking in the gastrocnemius following 2–4 weeks of microgravity exposure and there being no significant difference in the weight of the gastrocnemius between flight and ground control mice by week eight. Our observation of statistically significant atrophy in the gastrocnemius following 9 weeks of microgravity exposure is likely indicative of our larger sample size (*n* = 10 versus *n* = 5) and/or our use of a different method for measurement of atrophy (CSA reduction versus weight reduction). Consistent with our findings, atrophy has been identified in both slow-twitch and fast-twitch muscles as well as both flexors and extensors after as long as 13 weeks of microgravity exposure [99]. In contrast to the time-course nature of Cadena et al. [109]’s work, our study and most others [19, 20, 98, 99] employ a single, terminal timepoint for sample collection. This is a systemic limitation of microgravity research, such that acute and dynamic adaptations to microgravity are often unappreciated due to the limited capacity for in-flight sample collection, handling, processing, and storage, especially when studying tissues that require euthanasia for collection, such as skeletal muscle. Even time-course studies like Cadena et al. [109] are limited by small sample sizes and non-acute timepoints, such that what appeared to be potentially dynamic trends in expression of *MuRF1* and *MAFBx*, two well-characterized atrophy genes, in the gastrocnemius across 1, 2, 4, and 8 weeks of microgravity exposure were unsupported statistically. Therefore, there is a definite need for similar studies using larger samples and earlier timepoints. Our hope would be that the work presented here would provide ample justification for investigation of acute and dynamic changes not only in DGE but also in DAS in skeletal muscle across prolonged spaceflight.

In addition to the design-based limitations discussed above, there are variables independent of our study design that may have impacted its results, including potential behavioral differences between spaceflown mice and ground controls. There are no formal behavioral analyses available from the Rodent Research-5 (RR-5) mission of which our mice were a part. However, Ronca et al. [110] analyzed the behavior of mice from the Rodent Research-1 (RR-1) mission that utilized the same NASA Rodent Research Hardware System as RR-5. Ronca et al. [110] found that both time spent feeding and post-flight body weight were comparable across flight and ground control mice. While we have no information regarding food intake during RR-5, we can report that flight mice of a different cohort than those used in this study were of comparable weight to ground control mice when measured 24 h after live return to Earth. Therefore, food intake was likely similar across flight and ground control groups, and as such, this variable is not expected to have impacted our results. In addition, Ronca et al. [110] observed high levels of distinctive circling or “race tracking” behavior in younger mice (16 weeks old at launch) but minimal race tracking in older mice (32 weeks old at launch). Although race tracking was not formally analyzed during RR-5, observations of video taken during RR-5 suggest that, similar to Ronca et al. [110]’s findings, our 30-week-old mice exhibited some but not a lot of race tracking behavior. Therefore, activity patterns were likely somewhat dissimilar across flight and ground control groups, and as such, this variable is expected to have impacted our results, albeit minimally. Considering this activity pattern would have activated the muscles analyzed here during microgravity, the magnitude of the phenotypes we observed was likely attenuated as compared to what would be expected in the absence of race tracking during spaceflight.

## Conclusions

In summary, we have shown that following 9 weeks of spaceflight (i) DAS and DGE varied in a reciprocal manner, possibly in response to tissue-specific energy availability, (ii) transcripts encoding skeletal muscle proteins were primarily differentially spliced while non-differentially expressed, suggesting a more prominent role for DAS than DGE in regulating the transcriptomic response of hind limb muscles to microgravity, (iii) DAS events were associated with the physiological changes to the gastrocnemius and quadriceps in microgravity, including muscle atrophy and the potential expansion of fast-twitch functional capacity, and (iv) RBPs, *trans*-regulators of DAS, were themselves differentially spliced while being non-differentially expressed. Together, the results of our work emphasize the importance of DAS in determining the plasticity and functional status of the skeletal muscle transcriptome in microgravity. This knowledge is significant because it allows for identification of new potential targets for therapeutic intervention. Specifically, various small molecule splicing regulators have been recently approved for the treatment of atrophic neuromuscular diseases such as spinal muscle atrophy and muscular dystrophy. These therapeutics act as splice-switching oligonucleotides that foster the inclusion of an exon (*SMN* exon 7, nusinersen [111]) or skipping of an exon (*DMD* exon 51, eteplirsen [112]; *DMD* exon 53, golodirsen [113] and viltolarsen [114]), resulting in proteins with better functionality, associated with improvement in signs and symptoms of muscle disease. Ultimately, further characterization of microgravity-induced DAS will guide the search for small molecule splicing modulator-based therapies that mitigate microgravity-induced muscle atrophy, fiber type alterations, and other affiliated, detrimental physiological adaptations to prolonged spaceflight.

## Supplementary Information

**Additional file 1. **Complete list of DGE genes in the gastrocnemius. 120 genes were significantly differentially expressed across spaceflight in the gastrocnemius. Reported here are the gene symbol, log_2_ fold change (log_2_ FC), and FDR-adjusted *p*-value as reported by DeSeq2. Genes are ordered by significance of DGE as measured by FDR.

**Additional file 2. **Complete list of DGE genes in the quadriceps. 70 genes were significantly differentially expressed across spaceflight in the quadriceps. Reported here are the gene symbol, log_2_ fold change (log_2_ FC), and FDR-adjusted *p*-value as reported by DeSeq2. Genes are ordered by significance of DGE as measured by FDR.

**Additional file 3. **Complete list of DAS events in the gastrocnemius. 159 DAS events in 72 genes were identified across spaceflight in the gastrocnemius. Reported here are the gene symbol, event type (SE, skipped exon; MXE, mutually exclusive exons), △PSI (change in percent spliced in of a specific exon), FDR-adjusted *p*-value as reported by rMATS-turbo, and genome coordinates. Events are ordered first by gene symbol in alphabetical order and then by significance of DAS as measured by FDR.

**Additional file 4. **Complete list of DAS events in the quadriceps. 285 DAS events in 180 genes were identified across spaceflight in the quadriceps. Reported here are the gene symbol, event type (SE, skipped exon; MXE, mutually exclusive exons), △PSI (change in percent spliced in of a specific exon), FDR-adjusted *p*-value as reported by rMATS-turbo, and genome coordinates. Events are ordered first by gene symbol in alphabetical order and then by significance of DAS as measured by FDR.

**Additional file 5. **Gene ontology analyses. Enrichment by gene ontology of upregulated (first), downregulated (second), and alternatively spliced (third) genes in the gastrocnemius (left column) and quadriceps (right column). Reported here are the GO term [top five for each category (biological process, molecular function and cellular component)] and adjusted *p*-value. Asterisks represent significance as reported by EnrichR.

**Additional file 6: Figure S1.** RNA-seq quality control. **A** Summary of read depth (left axis with corresponding bar graph) and mapping statistics (right axis with corresponding line graph) for each RNA-seq dataset. Datasets are labeled by condition (Flight vs Ground), replicate (01, 02, 03), muscle type (g, gastrocnemius; q, quadriceps), and mouse identification number (M##). **B** Summary table of AS events detected by rMATS-turbo after filtering by read coverage and PSI value range. SE, skipped exon; A5SS, alternative 5’ splice site; A3SS, alternative 3’ splice site; MXE, mutually exclusive exons; RI, retained intron. Representative images below depict examples of the above listed alternative splicing events. Lines connecting exons represent splicing junctions, dark regions represent constantly retained transcript regions, and light regions represent alternatively spliced regions that are either included or excluded based on chosen splicing pattern. **Figure S2.** Fiber type patterns at low magnification. **A** Using AEC (3-Amino-9-Ethylcarbazole) staining at low magnification, we confirmed the fiber type distribution patterns of the gastrocnemius and quadriceps in ground control mice. **B** Representative immunohistochemistry images are also provided of gastrocnemius stained for MyHC I in ground control and flight mice, confirming the spaceflight-induced reduction in MyHC I expression in this muscle.

## Data Availability

The data that support the findings of this study are openly available in Gene Expression Omnibus at https://www.ncbi.nlm.nih.gov/geo/query/acc.cgi?acc=GSE178822.

## Abbreviations

DGE: Differential gene expression
DAS: Differential alternative splicing
MyHC: Myosin heavy chain
NASA: National Aeronautics and Space Administration
RBP: RNA-binding protein
AS: Alternative splicing
SE: Skipped exon
MXE: Mutually exclusive exons
RNA-seq: RNA-sequencing
PBS: Phosphate-buffered saline
BMD: Bone mineral density
KSC: Kennedy Space Center
ISS: International Space Station
CRS: Commercial Resupply Service
TERM: Terminal
UCLA: University of California, Los Angeles
CSA: Cross-sectional area
TCGB: Technology Center for Genomics and Bioinformatics
PSI: Percent spliced in of a specific exon
A5SS: Alternative 5′ splice site
A3SS: Alternative 3′ splice site
RI: Retained intron
GO: Gene ontology
FDR: False discovery rate
AEC: 3-Amino-9-ethylcarbazole
FC: Fold change
△PSI: Change in percent spliced in of a specific exon
UBA: Ubiquitin-associated
UBL: Ubiquitin-like
PEVK: Pro-Glu-Val-Lys
SVR: Splicing variable region
Ca^2+^: Calcium
ZF: Zinc finger
B40: Brain-specific exon consisting of 40 base pairs
M43: Muscle-specific exon consisting of 43 base pairs
TSS: Translational start site
RR5: Rodent Research-5
RR1: Rodent Research-1
