# Supplementary material for: Alternative splicing diversifies the skeletal muscle transcriptome during prolonged spaceflight
Source: Skelet Muscle. 2022 May 31;12:11. doi: 10.1186/s13395-022-00294-9 (PMC9153194; doi:10.1186/s13395-022-00294-9)
Supplement: Supplementary file 6 — Additional file 6: Figure S1. RNA-seq quality control. A Summary of read depth (left axis with corresponding bar graph) and mapping statistics (right axis with corresponding line graph) for each RNA-seq dataset. Datasets are labeled by condition (Flight vs Ground), replicate (01, 02, 03), muscle type (g, gastrocnemius; q, quadriceps), and mouse identification number (M##). B Summary table of AS events detected by rMATS-turbo after filtering by read coverage and PSI value range. SE, skipped exon; A5SS, alternative 5’ splice site; A3SS, alternative 3’ splice site; MXE, mutually exclusive exons; RI, retained intron. Representative images below depict examples of the above listed alternative splicing events. Lines connecting exons represent splicing junctions, dark regions represent constantly retained transcript regions, and light regions represent alternatively spliced regions that are either included or excluded based on chosen splicing pattern. Figure S2. Fiber type patterns at low magnification. A Using AEC (3-Amino-9-Ethylcarbazole) staining at low magnification, we confirmed the fiber type distribution patterns of the gastrocnemius and quadriceps in ground control mice. B Representative immunohistochemistry images are also provided of gastrocnemius stained for MyHC I in ground control and flight mice, confirming the spaceflight-induced reduction in MyHC I expression in this muscle. [file 13395_2022_294_MOESM6_ESM.docx]

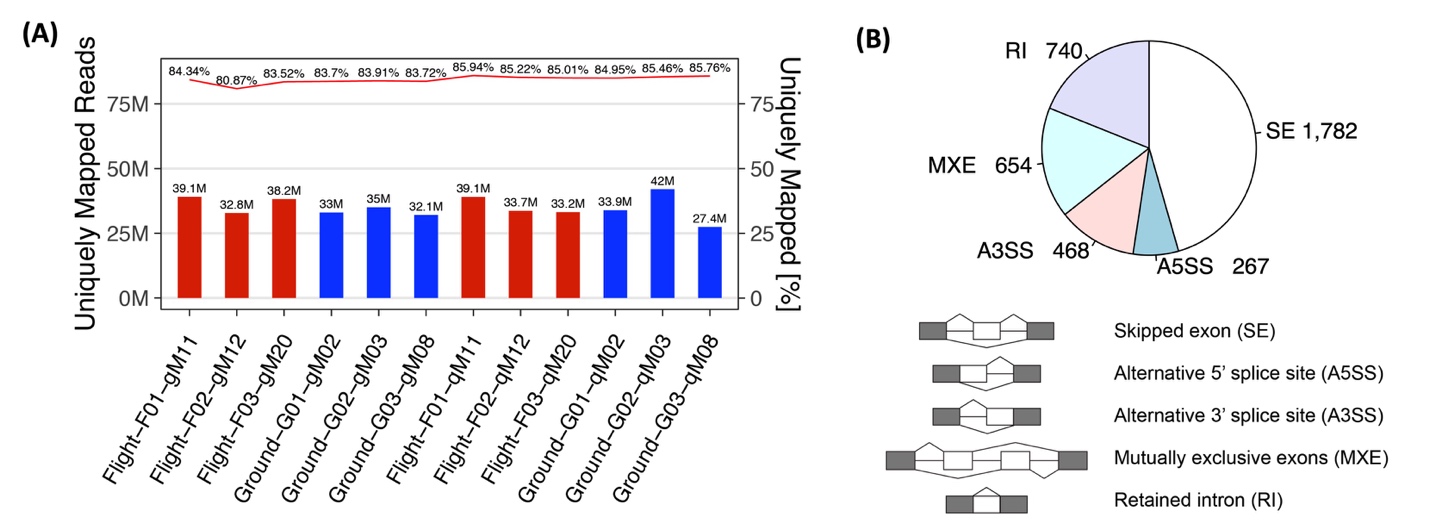


**Figure S1. RNA-seq quality control. (A)** Summary of read depth (left axis with corresponding bar graph) and mapping statistics (right axis with corresponding line graph) for each RNA-seq dataset. Datasets are labeled by condition (Flight vs Ground), replicate (01, 02, 03), muscle type (g, gastrocnemius; q, quadriceps), and mouse identification number (M##). **(B)** Summary table of AS events detected by rMATS-turbo after filtering by read coverage and PSI value range. SE, skipped exon; A5SS, alternative 5’ splice site; A3SS, alternative 3’ splice site; MXE, mutually exclusive exons; RI, retained intron. Representative images below depict examples of the above listed alternative splicing events. Lines connecting exons represent splicing junctions, dark regions represent constantly retained transcript regions, and light regions represent alternatively spliced regions that are either included or excluded based on chosen splicing pattern.

**
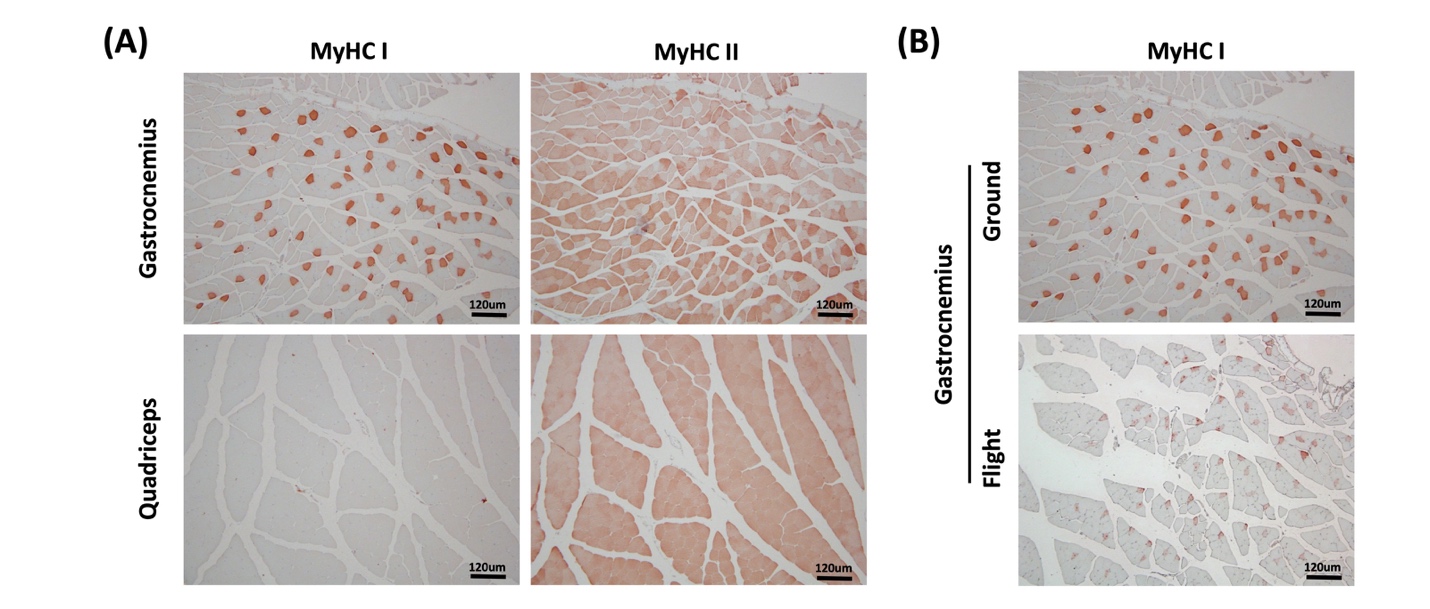
**

**Figure S2. Fiber type patterns at low magnification. (A)** Using AEC (3-Amino-9-Ethylcarbazole) staining at low magnification, we confirmed the fiber type distribution patterns of the gastrocnemius and quadriceps in ground control mice. **(B)** Representative immunohistochemistry images are also provided of gastrocnemius stained for MyHC I in ground control and flight mice, confirming the spaceflight-induced reduction in MyHC I expression in this muscle.
